# Therapy-imprinted fibroblast memory in cholangiocarcinoma: rewiring CAF niches for immune evasion and treatment-resistant relapse

**DOI:** 10.3389/fimmu.2026.1907838

**Published:** 2026-07-22

**Authors:** Longhao Zhang, Kai Zhang, Zhihong Chen, Xin Lu

**Affiliations:** Department of Liver Surgery, Peking Union Medical College Hospital, Chinese Academy of Medical Sciences and Peking Union Medical College, Beijing, China

**Keywords:** cancer-associated fibroblasts, cholangiocarcinoma, extracellular matrix, immune exclusion, senescence, spatial transcriptomics, therapeutic resistance, therapy-imprinted memory

## Abstract

Cholangiocarcinoma (CCA) is a highly desmoplastic biliary malignancy in which durable benefit from chemotherapy, immune checkpoint blockade, targeted therapy and local treatment remains limited for many patients. Cancer-associated fibroblasts (CAFs) are increasingly recognized as dynamic regulators of immune escape and therapeutic resistance rather than passive matrix-producing bystanders. Here, we propose therapy-imprinted fibroblast memory as a framework for understanding how treatment-conditioned CAF states may persist after therapeutic or tissue-injury pressure and contribute to immune exclusion, residual tumor-cell survival and relapse in CCA. This concept is distinguished from baseline CAF heterogeneity and transient stromal activation by prior therapeutic exposure, persistence beyond the acute injury phase, measurable molecular or spatial signatures, functional effects on tumor or immune behavior, and potential reversibility or targetability. Throughout the review, CCA-specific studies are prioritized to define disease-relevant CAF states, hepatobiliary injury contexts and therapeutic pressures, whereas evidence from pancreatic cancer, breast cancer, colorectal cancer, lung cancer, fibrosis and wound-healing models is used as mechanistic support for conserved stromal biology and hypothesis generation rather than as direct proof of therapy-imprinted CAF memory in CCA. We first summarize the established baseline CAF landscape in treatment-naive CCA, including myofibroblastic, inflammatory, antigen-presentation-like, perivascular and FAP-positive immunoregulatory CAF states. We then discuss how cytotoxic therapy, immune pressure, targeted therapy, radiotherapy, hypoxia, bile-acid stress, cholestasis and wound-healing signals may reshape these baseline programs into persistent post-treatment CAF niches. We further describe how therapy-conditioned CAF programs may reorganize spatial niches at invasive fronts, fibrotic septa, perivascular regions, perineural compartments and immune-excluded tumor borders, thereby supporting residual tumor cells, restricting cytotoxic lymphocyte access and promoting treatment-resistant relapse. Finally, we outline biomarker and therapeutic strategies for identifying and rewiring pathogenic CAF niches using single-cell and spatial multiomics, multiplex imaging, patient-derived models, FAP imaging, senescence-associated interventions, ECM normalization and rational stromal-immunotherapy combinations. This framework emphasizes that post-treatment CAF evolution should be interpreted against the baseline CCA CAF landscape rather than treated as an extension of static CAF taxonomy alone.

## 1 Introduction

Cholangiocarcinoma (CCA) is an anatomically, molecularly and clinically heterogeneous biliary malignancy for which late diagnosis, frequent recurrence and limited long-term disease control remain major clinical barriers (1). Intrahepatic, perihilar and distal CCA differ in risk factors, genomic profiles, stromal architecture and therapeutic vulnerabilities, supporting the need to interpret malignant evolution within its anatomical and microenvironmental context (2). Even after potentially curative resection, relapse is common, and most patients with advanced disease eventually experience progression despite systemic therapy (3). These features have made CCA a disease in which tumor-cell evolution and desmoplastic stromal adaptation should be considered together rather than as independent processes (4).

Systemic therapy for advanced biliary tract cancer has improved, but durable responses remain limited. Gemcitabine plus cisplatin established the first-line chemotherapy backbone, second-line FOLFOX provided survival benefit after platinum-gemcitabine progression, and adjuvant capecitabine became widely adopted after BILCAP in resected disease (5–7). More recently, TOPAZ-1 and KEYNOTE-966 showed that adding durvalumab or pembrolizumab to gemcitabine plus cisplatin improves outcomes in advanced biliary tract cancer (8, 9). These regimens are important not only as therapeutic advances, but also as sources of cytotoxic, inflammatory and tissue-repair pressure that may reshape both malignant and non-malignant compartments.

Precision oncology has further diversified the therapeutic landscape in molecularly selected CCA. Ivosidenib improved outcomes in previously treated IDH1-mutant CCA, while FGFR2 inhibitors such as pemigatinib and futibatinib produced clinically meaningful responses in FGFR2 fusion or rearranged intrahepatic CCA (10–13). HER2-directed therapy and BRAF/MEK inhibition have extended targeted options to additional molecular subsets, yet acquired resistance remains common (14, 15). This clinical pattern is consistent with genomic and epigenomic studies showing that CCA contains recurrent alterations in IDH1/2, FGFR2, BAP1, ARID1A, TP53, KRAS and ERBB2, as well as etiologically distinct molecular classes with divergent mutational, methylation and inflammatory features (16–19).

Although many resistance mechanisms are tumor-cell intrinsic, increasing evidence indicates that the CCA tumor microenvironment can determine whether treatment-induced immune or cytotoxic pressure is translated into durable tumor control. Genomic studies have highlighted therapeutically relevant kinase pathways and broader driver landscapes in biliary tract cancer, but these alterations operate within stromal and immune ecosystems rather than in isolation (20, 21). Immune-subtype analyses have shown that intrahepatic CCA contains distinct inflammatory and stromal microenvironments, and experimental CCA models indicate that suppressive myeloid populations can constrain PD-1 blockade responses (22, 23). The desmoplastic CCA stroma may also limit drug penetration, lymphocyte infiltration and productive immune-cell contact with malignant ducts, while tumor-stemness programs suggest that microenvironmental plasticity participates in recurrence and resistance (24, 25).

This review focuses on CAFs as dynamic stromal regulators of post-treatment CCA biology. The central premise is that baseline CAF heterogeneity and therapy-conditioned CAF persistence should be interpreted together but not conflated. Treatment-naive CCA studies define the baseline stromal landscape, including matrix-producing, inflammatory, perivascular, antigen-presentation-like and FAP-positive immunoregulatory CAF states (26–33). Post-treatment and recurrent settings then provide the context in which these baseline programs may be selected, stabilized or spatially reorganized by chemotherapy, immune checkpoint blockade, molecular targeted therapy, radiotherapy, locoregional therapy, cholestasis and tissue repair (5, 8–15, 33–35). To avoid conflating direct disease evidence with cross-cancer inference, this review uses an explicit evidence hierarchy. CCA-specific studies are used as the primary evidence base for baseline CAF states, biliary injury context, bile-acid-driven stromal activation, CAV1-related CAF senescence, extracellular-vesicle-mediated tumor-CAF crosstalk, stromal chemoresistance and CCA therapeutic pressures (1, 2, 26, 29, 33–37). Evidence from pancreatic cancer, breast cancer, colorectal cancer, lung cancer, fibrosis and wound-healing models is used as mechanistic support for conserved CAF biology, spatial organization, senescence, ECM remodeling and stromal immune regulation, but is not presented as direct proof that the same therapy-imprinted CAF states already operate in CCA (38–44). Using this structure, we propose therapy-imprinted fibroblast memory as a disease-relevant framework in which persistent CAF states contribute to immune exclusion, residual tumor-cell survival and treatment-resistant relapse.

## 2 Baseline CAF taxonomy versus therapy-imprinted fibroblast memory

Baseline CAF heterogeneity and therapy-imprinted fibroblast memory describe related but distinct aspects of stromal biology. Baseline CAF heterogeneity refers to the fibroblast state-space present before treatment, shaped by anatomical subtype, hepatic stellate cell contribution, portal or periductal fibroblast origin, cholestasis, bile-acid exposure, chronic inflammation and tumor genotype (1, 2, 26, 33, 45). Therapy-imprinted fibroblast memory refers to a persistent post-exposure CAF state generated or stabilized by therapeutic or tissue-injury pressure. In this review, a CAF state is considered therapy-imprinted when it is associated with prior therapeutic or injury-related pressure, persistence beyond the acute injury or wound-healing phase, a measurable molecular or spatial signature, functional consequences for tumor-cell survival, immune-cell localization, drug delivery or relapse behavior, and potential reversibility or targetability through stromal, immune or metabolic intervention (41, 42, 46).

Fibroblasts perform tissue-specific roles in development, repair, inflammation and matrix homeostasis, and their transformation into CAFs can remodel nearly every hallmark of cancer (47). A consensus framework has argued that CAF research should integrate origin, marker expression, location, function and clinical context rather than relying on single markers (45). Translational reviews have similarly emphasized that CAFs can be therapeutically relevant but heterogeneous, with both tumor-promoting and tumor-restraining functions (48). This duality is central for CCA, because indiscriminate stromal depletion may be harmful whereas niche-specific reprogramming may be beneficial.

Modern CAF biology distinguishes myofibroblastic, inflammatory, antigen-presenting, vascular-associated and matrix-remodeling states across tumor types (38). The field has also recognized that CAF origins may include resident fibroblasts, stellate cells, pericytes, mesenchymal stromal cells and transition-like programs, with lineage and tissue context shaping function (49). Because CAF markers such as ACTA2, FAP, PDGFRB, PDPN, COL1A1 and POSTN are not universally specific, marker panels and spatial context are needed for interpretation (39). In this review, we therefore treat CAF identity as a state-space rather than a fixed lineage label.

For nomenclature, this review uses myCAF for myofibroblastic or matrix-producing CAF states, iCAF for inflammatory cytokine-producing CAF states, apCAF-like for MHC-II/CD74-associated antigen-presentation-like programs and vCAF-like for perivascular or vascular-associated fibroblast programs. FAP-positive immunoregulatory CAF states are not treated as an independent lineage. Instead, FAP positivity is described as an overlapping functional and targetable state that may coexist with myCAF or iCAF programs depending on spatial location, cytokine exposure and treatment context. This terminology is used consistently throughout the manuscript to avoid implying that single markers define stable CAF lineages (28–32, 38, 39, 45, 49).

CAFs orchestrate malignant behavior by integrating soluble factors, extracellular matrix, mechanics and immune regulation (50). Pancreatic cancer studies first formalized the distinction between inflammatory CAFs and myofibroblastic CAFs, showing that CAF subtype depends on tumor-derived signals and spatial proximity (51). Antigen-presenting CAFs were subsequently identified in pancreatic cancer and shown to express MHC class II-related programs, expanding the functional repertoire of CAF subsets (52). A complementary study showed that IL-1 and TGF-beta can antagonistically shape inflammatory and myofibroblastic CAF states, suggesting that treatment-induced cytokine balance may shift CAF composition (53).

In human cancers, CAF states can correlate with immunotherapy outcome. LRRC15-positive myofibroblasts identified by single-cell RNA sequencing were associated with poor response to immune checkpoint blockade, supporting the concept that specific stromal states rather than total stromal abundance may predict immunotherapy resistance (54). Breast cancer studies have shown that CAF-S1-like populations can promote immunosuppressive T-cell ecosystems (55). CAF composition also changes during breast cancer progression, linking stromal subtype ratios to clinical outcome (56). Lung cancer single-cell analysis further demonstrated that stromal-cell phenotypes are molded by the tumor microenvironment in a tissue-specific manner (57).

Single-cell ecosystem studies in head and neck cancer showed that fibroblast and immune states can be organized with tumor-cell programs in recurrent neighborhoods (58). Spatially resolved and single-cell CAF studies in breast cancer identified functionally distinct fibroblast subclasses, reinforcing the need to connect CAF taxonomy with tissue geography (59). Cross-tissue fibroblast atlases suggest that fibroblast lineages have conserved organizational principles across organs while retaining tissue-specific modules (60). Single-cell criteria for separating fibroblasts from mural cells have improved annotation rigor and are directly relevant to identifying vascular CAF-like states in CCA (61). Liver fibrosis single-cell studies show zonated mesenchymal functions, which is important because CCA arises in a hepatic and biliary environment already shaped by injury and fibrogenesis (62). Finally, pancreatic fibroblast lineage studies have reminded the field that some CAF programs may support anti-tumor immunity, cautioning against broad CAF ablation (40).

These non-CCA studies are included to define conserved principles of CAF plasticity, spatial organization and immune regulation, but they should not be interpreted as direct evidence of therapy-imprinted CAF memory in CCA (38–43). In the present framework, cross-cancer evidence supports biological plausibility and experimental design, whereas CCA-specific studies define the disease-relevant stromal reference state and determine whether a proposed CAF program is applicable to cholangiocarcinoma (26, 29, 33, 35). Therefore, pancreatic, breast, lung, colorectal, fibrosis and wound-healing data are used as mechanistic comparators, while treatment-naive and post-treatment CCA tissues remain the required validation context for disease-specific conclusions.

## 3 Baseline CAF landscape in treatment-naive cholangiocarcinoma

CCA is one of the most desmoplastic epithelial malignancies, and early conceptual work highlighted the role of cancer-associated myofibroblasts in intrahepatic CCA progression (63). The desmoplastic stroma of CCA was later framed as a clinically relevant therapeutic target because it influences tumor-cell survival, invasion, perfusion and drug delivery (64). Reviews focused on CCA CAFs have summarized evidence that activated fibroblasts are major components of the CCA stroma and interact extensively with malignant cholangiocytes (26). More recent synthesis has emphasized that CAF biology in CCA includes ECM deposition, angiogenesis, lymphangiogenesis, cytokine signaling and therapeutic resistance (27). A 2025 CCA CAF review also summarized subtype markers such as myCAF, iCAF, apCAF-like and vCAF-like features, demonstrating that the baseline field is now sufficiently mature for a more specialized post-treatment framework (28).

Most CCA CAF studies to date describe treatment-naive or surgically resected primary tumors. These studies are essential for defining the baseline stromal reference state but should not be interpreted as direct evidence of therapy-imprinted memory. For example, myCAF-like matrix programs, iCAF-like inflammatory programs, apCAF-like MHC-II/CD74-associated states, vCAF-like perivascular programs and FAP-positive immunoregulatory states can all exist before systemic therapy (26–33). Therefore, throughout this review, baseline CAF states are treated as the reference landscape against which post-treatment CAF evolution should be measured.

A landmark CCA study demonstrated that diverse CAF subpopulations promote intrahepatic CCA growth and that hepatic stellate cells contribute substantially to CAF generation in experimental models (29). Single-cell transcriptomics of human intrahepatic CCA identified intercellular crosstalk between malignant cells and stromal populations, including vascular CAF-like programs influenced by tumor-derived extracellular vesicles (30). Microenvironment-based classification of intrahepatic CCA further showed that stromal and immune architecture can stratify tumors with therapeutic implications (31). Independent single-cell analysis supported the existence of distinct intrahepatic CCA molecular and microenvironmental phenotypes (32).

Mechanistic CCA studies have linked CAFs to receptor tyrosine kinase and survival signaling. Hepatic myofibroblasts can promote CCA progression through EGFR activation in malignant cells (65). Myofibroblast-derived PDGF-BB can protect CCA cells through Hedgehog-dependent survival signaling (66). PDGF-D and Rho GTPase signaling can regulate recruitment of CAFs to the CCA microenvironment (67). The stromal-derived factor-1/CXCR4 axis can mediate CCA-stromal interaction and is therefore a plausible mechanism for chemokine-based immune exclusion (36).

CCA-stromal crosstalk also intersects with EMT-like invasion. Angiotensin II has been reported to enhance epithelial-to-mesenchymal transition through interactions between CCA cells and hepatic stellate cells (37). Experimental deletion of CAFs in a CCA model produced therapeutic effects, showing that at least some CAF compartments actively sustain tumor progression (68). CAF-derived PDGF-D can promote lymphangiogenesis through VEGF-related programs, linking fibroblast activity to dissemination (69). Tumor-associated angiogenesis and lymphangiogenesis correlate with progression in intrahepatic CCA, supporting vascular and lymphatic niche relevance (70).

The tumor-reactive stroma of CCA has been described as the fuel behind aggressiveness because it envelopes malignant ducts in a fibrotic, hypovascular and inflammatory microenvironment (71). Reviews of the CCA immune milieu emphasize that CAFs, macrophages, endothelial cells and lymphocytes form a tightly coupled ecosystem rather than isolated compartments (72). SERPINE1/PAI-1 has recently been discussed as both a mediator of CCA progression and a potential stromal therapeutic target within the desmoplastic microenvironment (73). CAV1-related CAF senescence has been associated with tumor-infiltrating lymphocytes and PD-L1 expression in intrahepatic CCA, providing a direct link between senescent stromal features and immune architecture (34).

New evidence also implicates biliary metabolites in stromal activation. A 2025 Cancer Cell study reported that bile acids can activate CAFs and induce an immunosuppressive CCA microenvironment, which is particularly relevant because cholestasis and bile-acid stress are disease-contextual features rather than generic cancer signals (33). Extracellular vesicles released by bile duct cancer cells can activate CAFs through TGF-beta-dependent mechanisms, providing a mechanism for tumor-cell instruction of stromal memory (74). Extracellular vesicle delivery of miR-195 has been studied in intrahepatic CCA models, illustrating the broader relevance of vesicle-mediated communication in this disease (75). Autocrine and paracrine mechanisms of chemoresistance in CCA further support the premise that therapy resistance emerges from multicellular signaling rather than tumor-cell mutation alone (35). Together, treatment-naive CCA studies define the baseline stromal landscape from which therapy-conditioned CAF states may emerge. Baseline myCAF, iCAF, apCAF-like, vCAF-like and FAP-positive immunoregulatory programs can exist before systemic therapy, but therapeutic pressure may alter their abundance, spatial arrangement, ligand-receptor communication, senescence state, metabolic program or ECM-remodeling activity (26, 29). Therefore, the key biological question is not whether CAF heterogeneity exists in CCA, but how existing CAF programs are selected, stabilized or rewired after chemotherapy, immune checkpoint blockade, targeted therapy, radiotherapy, locoregional therapy or surgery (5, 8, 9, 33–35). A summary of major CAF states, including myCAF, iCAF, apCAF-like, vCAF-like, senescent CAF and mesothelial-like CAF programs, is provided in **Table 1**. FAP-positive immunoregulatory CAF states are presented as overlapping functional states rather than independent lineages, because FAP expression may coexist with myofibroblastic, inflammatory or matrix-remodeling programs depending on spatial and treatment context (28, 38, 39, 45).

**Table 1:** CAF states and proposed relevance in therapy-imprinted CCA niches.

CAF state/program	Representative markers	Dominant functions	Potential therapy-imprinted role	Suggested assays
myCAF/matrix-producing CAF state	ACTA2, TAGLN, COL1A1, COL3A1, POSTN, FN1	Collagen deposition, contractility, matrix alignment, tissue stiffening	Maintains immune-exclusion rim and physical drug-delivery barriers after chemotherapy or radiotherapy	scRNA-seq, spatial transcriptomics, alpha-SMA/FAP IHC, collagen SHG, AFM/rheology
iCAF/inflammatory CAF state	IL6, LIF, CXCL12, CCL2, CXCL14, HGF	Cytokine secretion, tumor-cell survival, myeloid recruitment, STAT3 activation	Protects residual tumor cells and sustains inflammatory but immunosuppressive relapse niches	Cytokine panels, ligand-receptor inference, organoid-CAF co-culture, phospho-STAT3 staining
apCAF-like state	HLA-DRA, HLA-DPA1, CD74, CIITA-related genes	MHC-II-associated antigen presentation-like programs, immune education	May be remodeled by interferon and ICI exposure; function in CCA remains to be experimentally defined	scRNA-seq, spatial MHC-II/CD74 multiplex IF, T-cell co-culture
vCAF-like/perivascular CAF state	RGS5, MCAM, PDGFRB, CSPG4, IL6	Vascular support, perivascular signaling, angiogenesis, lymphangiogenesis	Creates perivascular refuge for residual tumor cells and routes for dissemination	Spatial mapping around CD31/LYVE1/PDPN vessels, vascular perfusion imaging
FAP-positive immunoregulatory CAF state overlapping with myCAF/iCAF	FAP, PDPN, CXCL12, TGFB1, SERPINE1, POSTN or IL6 depending on niche context	Immune exclusion, matrix remodeling and chemokine-gradient formation; not a distinct lineage based on FAP expression alone	Possible targetable state for FAP imaging or FAP-directed delivery when spatially linked to immune exclusion or stromal relapse niches	FAPI PET plus tissue FAP IHC, CXCL12/TGF-beta spatial profiling, co-localization with myCAF and iCAF markers
Senescent CAF	p16, p21, CAV1-related patterns, IL6, CXCL8, MMPs, SASP genes	Persistent inflammatory secretion, matrix remodeling, myeloid recruitment	Post-genotoxic therapy relapse niche; candidate for senomorphic or senolytic strategies	Senescence panels, SASP proteomics, beta-gal-associated assays, multiplex IF
Mesothelial-like/surface-associated CAF	MSLN, UPK1B, UPK3B, KRT19-low stromal-associated programs	Peritoneal/surface interaction, matrix and inflammatory programs	May contribute to peritoneal dissemination or surgical-relapse niches	Lineage-informed scRNA-seq, peritoneal metastasis spatial profiling

The listed CAF states and markers are representative rather than exclusive. CAF programs may overlap within the same tumor and may shift across treatment contexts. In particular, FAP-positive immunoregulatory CAF states are treated here as overlapping functional states rather than independent lineages separate from myCAF or iCAF programs.

CAF, cancer-associated fibroblast; CCA, cholangiocarcinoma; ECM, extracellular matrix; FAP, fibroblast activation protein; FN1, fibronectin 1; IHC, immunohistochemistry; myCAF, myofibroblastic cancer-associated fibroblast; iCAF, inflammatory cancer-associated fibroblast; apCAF-like, antigen-presentation-like cancer-associated fibroblast program; vCAF-like, vascular/perivascular cancer-associated fibroblast-like program; scRNA-seq, single-cell RNA sequencing; SHG, second harmonic generation.

## 4 Operational definition of therapy-imprinted fibroblast memory in the biliary injury context

We define therapy-imprinted fibroblast memory as a persistent, treatment-conditioned CAF state that emerges after a defined therapeutic or tissue-injury exposure, remains detectable beyond the acute injury or repair phase, carries a measurable molecular, spatial or functional signature, alters subsequent tumor-cell or immune-cell behavior, and is potentially reversible or targetable through stromal, immune, metabolic or mechanical intervention (26, 33–35, 41–43). This definition intentionally excludes the interpretation that any post-treatment stromal alteration automatically represents fibroblast memory. A CAF program should be considered therapy-imprinted only when five criteria are met or explicitly tested: first, a documented or inferable prior exposure, such as chemotherapy, immune checkpoint blockade, targeted therapy, radiotherapy, locoregional therapy, surgery, biliary obstruction or cholestatic injury; second, persistence beyond transient acute inflammation or wound repair; third, a reproducible molecular, epigenetic, proteomic, spatial or imaging signature; fourth, functional consequences for residual tumor-cell survival, immune-cell localization, drug delivery, matrix remodeling or relapse behavior; and fifth, evidence that the state is reversible, targetable or clinically informative (5, 8, 9, 44). These criteria distinguish therapy-imprinted CAF memory from descriptive post-treatment histology alone.

Several related processes should therefore be distinguished from therapy-imprinted fibroblast memory. Baseline CAF heterogeneity refers to pre-existing stromal diversity in treatment-naive CCA and does not itself prove therapy imprinting (26, 29, 32, 33). Transient CAF activation refers to short-lived inflammatory or repair responses that resolve after the acute injury phase. Wound-healing responses after surgery or locoregional therapy may contribute to fibroblast memory only if they persist, acquire measurable signatures and influence relapse biology (41, 76–78). Therapy-induced senescence is a candidate memory mechanism, but senescence markers alone are insufficient unless linked to persistent SASP activity, spatial niche remodeling and functional effects on residual tumor or immune cells (42, 79–82). Fibrosis-associated ECM remodeling similarly becomes a memory-like process only when matrix architecture stores durable biochemical or mechanical signals that continue to affect tumor behavior after the initiating stimulus has changed (43, 83). Ordinary adaptive resistance should also be separated from fibroblast memory unless CAF-derived programs are shown to contribute directly to residual-cell protection, immune exclusion or treatment escape (35, 65–67, 84, 85).

For clarity, the evidentiary strength of this concept is interpreted at three levels. First, CCA-specific evidence includes studies of CCA CAF subpopulations, hepatic stellate cell contribution, CCA stromal crosstalk, bile-acid-induced CAF activation, CAV1-related CAF senescence, extracellular-vesicle-mediated CAF education and CCA chemoresistance (35, 63). Second, hepatobiliary and fibrosis evidence provides disease-contextual support for how liver injury, cholestasis, inflammation and fibrogenesis can shape fibroblast behavior (1, 2, 33, 62, 71). Third, non-CCA cancer and wound-healing studies provide mechanistic support for conserved processes such as fibroblast memory, CAF plasticity, SASP, ECM stiffness, metabolic coupling and immune exclusion, but these data are treated as hypothesis-generating rather than disease-definitive for CCA (38, 40–44, 76, 86, 87). Fibroblast memory has been described in development, homeostasis and disease as the capacity of fibroblasts to retain information from prior positional, inflammatory or injury-associated experiences (41). Dermal lineage-tracing studies show that intrinsically fibrogenic fibroblast lineages can be prospectively isolated, implying that fibroblast origin can encode durable fibrotic capacity (88). Developmental studies have also shown that distinct fibroblast lineages determine dermal architecture and repair outcomes (89).

Wound-healing single-cell studies demonstrate that fibroblast populations are heterogeneous and dynamically remodeled after injury (90). Myofibroblast-to-adipocyte conversion during wound healing shows that fibroblast-derived states can be plastic rather than terminally fixed (76). Positional gene-expression programs in human fibroblasts provide a conceptual precedent for tissue-resident stromal memory (91). Epigenetic memory mechanisms may allow prior activation states to be retained through chromatin, enhancer or nuclear-architecture changes (92). Cancer epigenetics provides a broader framework for how stable transcriptional states can influence cellular phenotype and therapeutic response (93).

Human fibroblasts display topographic differentiation and positional memory across anatomical sites, which supports the idea that CCA-associated fibroblasts arising from portal, periductal or hepatic stellate sources may not respond identically to therapy (94). Clinical epigenetics has emphasized that epigenetic marks can be translated into biomarkers and therapeutic strategies, suggesting one route for detecting therapy-imprinted CAF memory in patient samples (95). In CCA, this concept is strengthened by the biliary injury environment, in which chronic liver injury, cholestasis, bile-acid exposure and inflammation may pre-prime fibroblasts before oncologic therapy begins (1, 2, 51). These disease-contextual pressures provide a CCA-specific background in which treatment-induced stress may not act on naive fibroblasts, but rather on fibroblast populations already shaped by biliary inflammation, fibrogenesis and tumor-stroma crosstalk (26, 32, 34, 35). Accordingly, when evidence is drawn from non-CCA systems, it is used to explain plausible mechanisms that should be tested in paired treatment-naive, post-treatment and recurrent CCA specimens, rather than to claim that the same CAF memory state has already been proven in CCA.

Senescence is another candidate storage system for therapy-imprinted CAF memory, but senescence alone should not be equated with fibroblast memory unless it persists after treatment and produces functional SASP-mediated effects on tumor, immune or matrix behavior (42, 46, 79–82). The senescence-associated secretory phenotype can convert a cell-cycle arrest program into a persistent inflammatory and tissue-remodeling signal (42). Cellular senescence links aging, cancer and tissue repair, and it can have both tumor-suppressive and tumor-promoting consequences depending on context (79). Senescent cells accumulate in age-related disease and are now considered therapeutic targets, making stromal senescence clinically relevant rather than merely descriptive (80). Chemotherapy-induced senescence can promote adverse treatment effects and cancer relapse, supporting a direct connection between therapy exposure and relapse-promoting secretory states (46).

**Figure 1:**
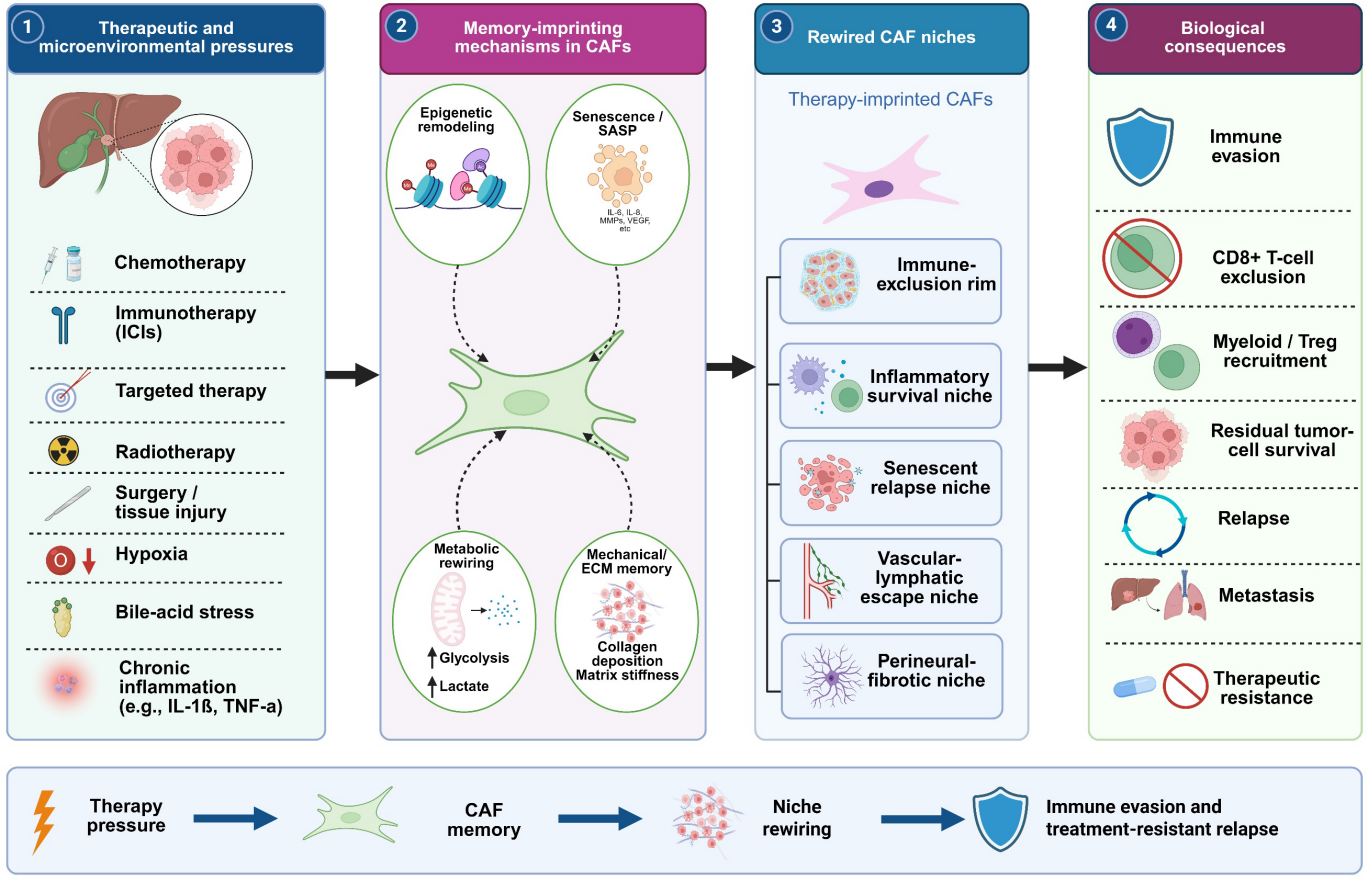
This conceptual diagram illustrates the formation and consequences of therapy−imprinted fibroblast memory in cholangiocarcinoma (CCA). The left side depicts multiple therapeutic and microenvironmental pressures, including chemotherapy, immunotherapy (immune checkpoint inhibitors, ICIs), targeted therapy, radiotherapy, surgery/tissue injury, hypoxia, bile−acid stress, and chronic inflammation (e.g., IL−1β, TNF−α). These stimuli converge on cancer-associated fibroblasts (CAFs) and are stored through interconnected memory-imprinting mechanisms: epigenetic remodeling (chromatin accessibility, DNA methylation, histone modifications), senescence and the senescence-associated secretory phenotype (SASP, involving p16/p21, IL-6, IL-8, MMPs, VEGF), metabolic rewiring (glycolysis, lactate, autophagy, redox adaptation), mechanical extracellular matrix (ECM) memory (collagen deposition, matrix stiffness, integrin-FAK-YAP/TAZ signaling), vesicle-mediated communication and biliary/cholestatic inflammatory crosstalk. In the CCA context, bile-acid stress, cholestasis and chronic biliary inflammation may connect these layers rather than acting as isolated upstream stimuli. The resulting therapy−imprinted CAFs reorganize the tumor microenvironment into distinct spatial niches (immune−exclusion rim, inflammatory survival niche, vascular-lymphatic escape niche, perineural−fibrotic niche, and senescent relapse niche). The final consequences are immune evasion (CD8^+^ T−cell exclusion, myeloid/Treg recruitment), residual tumor−cell survival, treatment−resistant relapse, and metastasis. This framework highlights that therapy acts not only on malignant cells but may also durably reprogram stromal cells to drive disease progression. However, a post-treatment CAF alteration should be interpreted as therapy-imprinted fibroblast memory only when it is linked to prior exposure, persistence beyond acute injury, a measurable molecular or spatial signature, functional effects on tumor or immune behavior and potential reversibility or targetability. CCA-specific evidence is used to define the disease context and stromal reference states, whereas non-CCA cancer, fibrosis and wound-healing evidence is used to illustrate conserved mechanisms that require validation in paired CCA specimens. CCA, cholangiocarcinoma; CAF, cancer−associated fibroblast; SASP, senescence−associated secretory phenotype; ECM, extracellular matrix; FAK, focal adhesion kinase; YAP/TAZ, Yes−associated protein/transcriptional coactivator with PDZ−binding motif.

Senescence-associated reprogramming can also promote cancer stemness, suggesting that therapy-induced stromal senescence might indirectly preserve stem-like residual CCA cells (96). Collectively, therapeutic pressures in CCA should be interpreted together with biliary-contextual stressors, including bile-acid exposure, cholestasis, chronic inflammation, hypoxia and tissue repair. However, these inputs should be considered memory-imprinting only when they generate CAF programs that persist beyond the acute exposure window, are measurable at molecular or spatial levels, and have demonstrable functional consequences for immune exclusion, residual tumor-cell survival, drug delivery or relapse-prone tissue remodeling (**Figure 1**) (33, 35, 42–44, 46, 86, 87). In liver cancer models, a senescence secretome can link metabolic and microbial factors to tumor promotion, providing a precedent for senescence-driven hepatobiliary oncogenesis (97). Senescent cells can mediate fibrotic pulmonary disease, showing that senescence and fibrosis can reinforce one another in non-malignant tissue injury (98). The broader role of senescent cells in ageing highlights why older CCA patients may possess stromal backgrounds that are more susceptible to therapy-induced memory formation (99). CXCR2-dependent chemokine reinforcement of senescence provides one example of how inflammatory chemokines can stabilize senescent phenotypes (100). Senolytic and senomorphic concepts provide potential interventions to test whether senescent CAF memory is reversible in CCA (101).

## 5 Integrated mechanical, metabolic and spatial storage of post-treatment CAF states in CCA

The extracellular matrix is a major structural and signaling reservoir for CAF memory. Matrix proteins modulate proliferation, invasion, angiogenesis, immune-cell trafficking and drug distribution in tumors (43). ECM assembly and remodeling operate across molecular, cellular and tissue scales, so post-treatment changes in collagen, fibronectin and hyaluronan can persist even after malignant-cell burden decreases (102). The ECM also functions as a dynamic niche that stores biochemical and mechanical signals during cancer progression (103).

Matrix crosslinking can force tumor progression by enhancing integrin signaling, providing a mechanism through which therapy-induced fibrosis may become self-sustaining (104). Tensional homeostasis connects tissue stiffness to malignant phenotype, implying that residual CCA cells within stiff post-treatment niches may receive survival cues even when oncogenic signaling is pharmacologically inhibited (105). YAP-dependent matrix remodeling is required for generation and maintenance of CAFs in experimental models, connecting mechanical feedback to fibroblast-state persistence (106). YAP/TAZ mechanotransduction more broadly explains how ECM stiffness can be translated into durable transcriptional programs (86).

ECM remodeling and homeostasis are central to both fibrosis and cancer, two processes that converge strongly in CCA (107). Matrix remodeling in development and disease also illustrates how proteases, crosslinking enzymes and matrix-associated growth factors can alter cell behavior long after the original stimulus (83). In human breast cancer, ECM stiffening correlates with invasion and immune-cell infiltration, supporting the translational relevance of mechanical biomarkers (108). Hyaluronan depletion studies in pancreatic cancer show that stromal mechanics can influence vascular perfusion and drug delivery, although such strategies require careful disease-specific validation (109). Vitamin D receptor-mediated stromal reprogramming in pancreatic cancer further illustrates that activated stroma can sometimes be normalized rather than eliminated (110).

Metabolic crosstalk provides another storage mode for therapy-imprinted CAF function. The reverse Warburg effect proposed that glycolytic stromal cells can fuel adjacent cancer cells, a concept that stimulated broader investigation of metabolic symbiosis in tumors (44). Stromal-epithelial metabolic coupling integrates autophagy and energy transfer within the tumor microenvironment (111). Tumor and stromal metabolism are bidirectional, and therapy-induced nutrient stress may select for CAF states that support residual tumor cells (112). CAF metabolic reprogramming can also contribute to tumor metabolic heterogeneity and therapeutic resistance (113).

Pancreatic stellate cells can support tumor metabolism through autophagic alanine secretion, offering a strong precedent for stellate-derived metabolic support in desmoplastic gastrointestinal tumors (114). Oncogenic KRAS signaling can regulate tumor-cell pathways through stromal reciprocation, suggesting that tumor genotype and CAF adaptation may form feedback loops (115). In CCA, stem-like tumor subsets can shape macrophage- and niche-related programs, linking metabolic plasticity, immune education and stromal support (116). Hypoxia-regulated extracellular vesicle biogenesis may further amplify post-treatment communication between stressed tumor cells and fibroblasts (87).

In CCA, epigenetic remodeling, senescence/SASP, metabolic rewiring and mechanical ECM feedback may converge through a biliary injury circuit rather than functioning as isolated modules. Bile-acid stress and cholestasis can activate inflammatory, oxidative-stress and TGF-beta-related stromal pathways, while hypoxia and therapy-induced tissue injury may reinforce DNA damage responses, SASP cytokine production and myeloid recruitment (33, 42, 46, 87). SASP mediators such as IL-6, CXCL8 and CCL2 may amplify inflammatory CAF programs and immune suppression, whereas collagen deposition, matrix crosslinking, integrin-FAK signaling and YAP/TAZ mechanotransduction can stabilize matrix-producing CAF states and restrict immune-cell access (42, 43, 84, 104). Metabolic stress, lactate exchange, autophagy and redox adaptation may further support residual tumor cells and impair cytotoxic immune function (62, 98, 117). This integrated circuit provides a CCA-centered rationale for linking biliary stress, therapy-conditioned CAF persistence, immune evasion and relapse.

Spatial technologies now make it possible to convert the concept of CAF memory into a testable anatomical framework. Spatial transcriptomics can map gene expression directly in tissue sections (118). Slide-seq provides high-resolution genome-wide spatial expression measurements (119). RNA seqFISH+ enables transcriptome-scale super-resolved imaging in tissues (117). Integration of spatial transcriptomics and single-cell RNA sequencing has revealed tissue architecture in pancreatic cancer, a model directly relevant to desmoplastic CCA (120). Digital spatial profiling allows multiplexed measurement of RNA and protein in fixed tissue, which is suitable for clinical specimens (121).

Although these technologies provide powerful discovery tools, spatial transcriptomics, CODEX, imaging mass cytometry and related multiplex platforms should not be presented as immediately deployable routine clinical assays for CCA. Major translational barriers include high cost, limited throughput, specialized instrumentation, dependence on advanced bioinformatics expertise, FFPE-associated RNA or protein degradation, variable fixation and tissue-processing protocols, incomplete antibody validation for multiplex imaging, batch effects across centers, inconsistent cell segmentation, non-uniform deconvolution pipelines and the lack of standardized cutoffs for clinically actionable spatial metrics (121–124). These issues are particularly relevant in CCA because biopsy specimens may be small, fibrotic, necrotic or anatomically heterogeneous across intrahepatic, perihilar and distal tumors (1, 2, 24, 64, 71).

A staged translational strategy should therefore proceed in phases. First, spatial multiomics can be used in retrospective and prospective correlative studies to define reproducible CAF-immune-tumor neighborhoods. Second, simplified multiplex immunofluorescence, imaging mass cytometry or IHC panels can be derived from spatial discovery data for FFPE-compatible validation. Third, locked analytical pipelines, predefined region-of-interest selection rules, minimum tissue-quality criteria, external validation cohorts and clinically interpretable spatial thresholds are required before spatial CAF biomarkers can guide patient selection. This staged approach preserves the value of spatial multiomics while acknowledging the practical barriers that currently limit routine clinical implementation (121, 123, 124).

**Figure 2:**
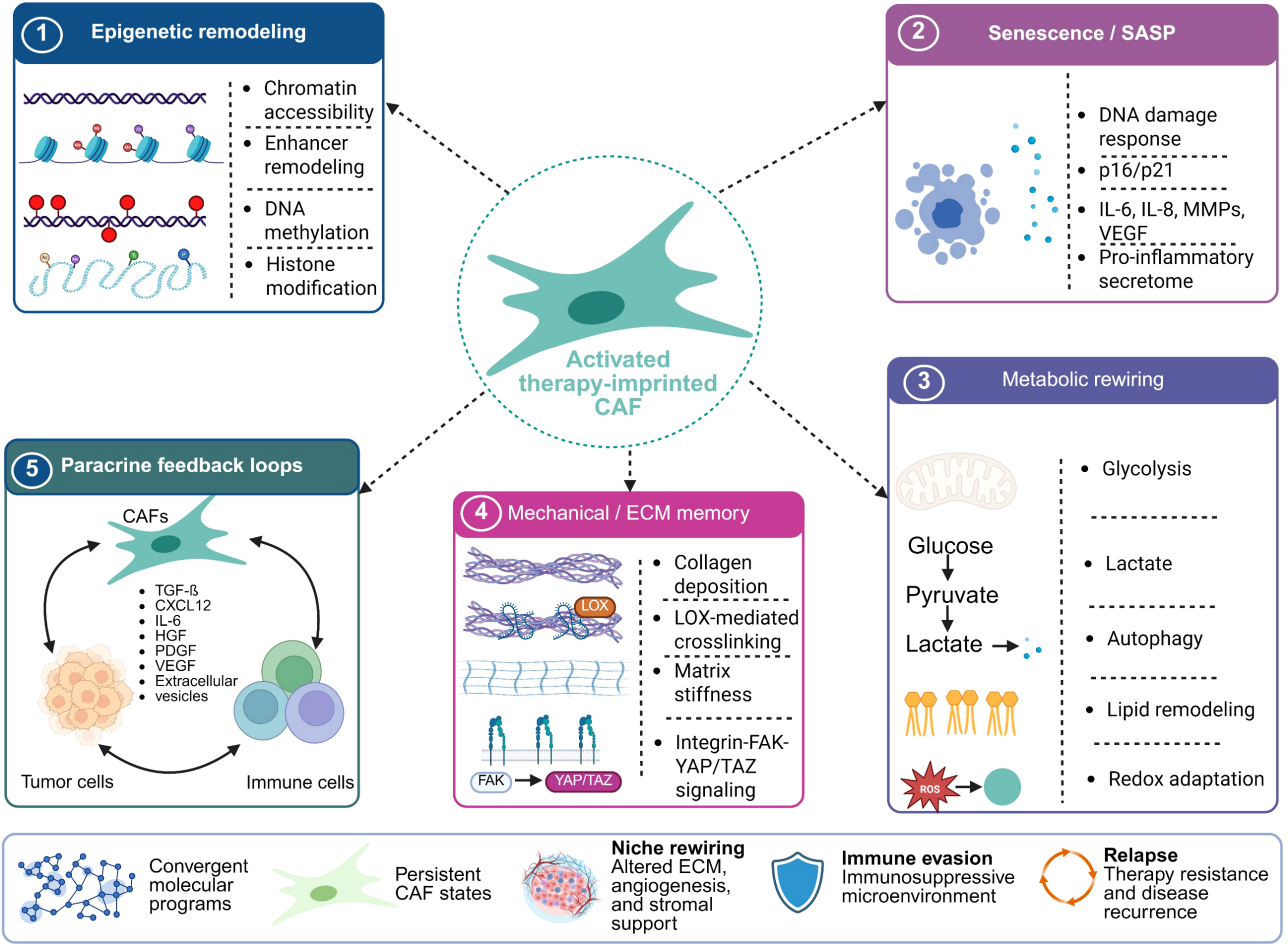
Molecular storage systems that may maintain therapy-imprinted CAF memory after treatment in CCA. This schematic summarizes interconnected mechanisms through which prior therapeutic or tissue-injury exposure may be stored in persistent CAF programs. First, epigenetic remodeling involves changes in chromatin accessibility, DNA methylation, histone modifications and enhancer activity, mediated by transcriptional regulators such as SMAD, NF-kappaB, STAT3 and AP-1, leading to sustained cytokine and ECM gene expression. Second, senescence and the SASP are triggered by DNA damage, oxidative stress, hypoxia and genotoxic therapy, inducing p16/p21 and a pro-inflammatory secretome including IL-6, IL-8, MMPs and VEGF. Third, metabolic rewiring includes glycolysis, lactate transport, autophagy, lipid remodeling and redox adaptation, creating metabolic support for residual tumor cells and potentially impairing T-cell function. Fourth, mechanical ECM memory involves collagen deposition, LOX-mediated crosslinking, integrin-FAK signaling and YAP/TAZ mechanotransduction, which sustain tissue stiffness and CAF activation after the initiating injury. Fifth, paracrine and vesicle-mediated feedback loops, including soluble factors such as TGF-beta, CXCL12, IL-6, HGF, PDGF and VEGF and extracellular vesicle cargo, propagate CAF activation to neighboring tumor and immune cells. In the CCA context, bile-acid stress, cholestasis, hypoxia and chronic biliary inflammation may connect epigenetic remodeling, SASP signaling, metabolic rewiring, vesicle-mediated communication and mechanical ECM feedback. These layers should be interpreted as candidate storage mechanisms rather than sufficient evidence of therapy-imprinted fibroblast memory in isolation; a post-treatment CAF program should be linked to prior exposure, persistence beyond acute injury, measurable molecular or spatial signatures, functional consequences and potential reversibility or targetability before being considered therapy-imprinted memory. CCA, cholangiocarcinoma; CAF, cancer-associated fibroblast; SASP, senescence-associated secretory phenotype; ECM, extracellular matrix; LOX, lysyl oxidase; FAK, focal adhesion kinase; YAP/TAZ, Yes-associated protein/transcriptional coactivator with PDZ-binding motif; NF-kappaB, nuclear factor kappa-B; AP-1, activator protein-1; HGF, hepatocyte growth factor; PDGF, platelet-derived growth factor; VEGF, vascular endothelial growth factor; MMP, matrix metalloproteinase.

CODEX multiplexed imaging can deeply profile tissue architecture and immune organization, but its clinical application in CCA requires standardized antibody panels, validated cell segmentation and reproducible spatial metrics before routine use (122). Cellular-neighborhood analysis at the colorectal cancer invasive front showed that spatially coordinated neighborhoods can orchestrate anti-tumor immunity (125). More recent colorectal cancer studies have further demonstrated that single-cell, spatial and systemic immune profiling can identify clinically relevant tumor-immune organization, stromal-immune interactions and macroenvironmental immune dysfunction (126, 127). These colorectal cancer studies provide methodological and conceptual support for spatial immune-neighborhood analysis, but CCA-specific spatial evidence is now also emerging. Single-cell and spatial transcriptomics of the intrahepatic CCA leading-edge area showed that leading-edge tumor cells are tightly associated with stromal components, including endothelial cells and POSTN+FAP+ fibroblasts (128). High-resolution spatial multi-omics has also been used to develop prognostic scoring in intrahepatic CCA, supporting the clinical relevance of spatially resolved CCA microenvironment analysis (129). In addition, a subcellular spatial atlas of distal CCA perineural invasion revealed coordinated epithelial, stromal and immune remodeling around nerve-associated structures (130). Together with conserved spatial CAF-neighborhood studies across cancers and FAP-positive CAF spatial analyses, these recent data strengthen the rationale for defining therapy-conditioned CAF niches in CCA using spatially resolved approaches (131, 132). The molecular programs that store therapy-imprinted CAF memory — including epigenetic remodeling, senescence/SASP, metabolic rewiring, mechanical ECM signaling, vesicle-mediated communication and biliary/cholestatic inflammatory crosstalk — operate as interconnected layers that may sustain post-treatment stromal dysfunction in CCA (**Figure 2**) (33–35, 42–44, 74, 75, 79, 86, 87). The candidate memory layers — including epigenetic remodeling, senescence/SASP, metabolic rewiring, mechanical ECM memory, vesicle-mediated communication, biliary/cholestatic inflammatory crosstalk and spatial niche organization — along with their triggers, mediators, functional outputs and reversibility hypotheses are outlined in **Table 2**. Importantly, these mechanisms represent candidate storage systems rather than diagnostic criteria by themselves; each layer must be linked to exposure history, persistence, spatial or molecular readout, functional consequence and reversibility or targetability before being interpreted as therapy-imprinted CAF memory.

**Table 2:** Mechanisms that may store therapy-imprinted fibroblast memory.

Memory layer	Likely triggers	Molecular mediators	Functional output	Reversibility hypothesis
Epigenetic memory	TGF-beta, IL-1, chemotherapy, radiotherapy, bile-acid stress, chronic inflammation	DNA methylation, histone marks, enhancer remodeling, SMAD, NF-kappaB, STAT3, AP-1	Persistent cytokine and ECM programs	Potentially reversible with chromatin, cytokine or pathway-level intervention
Senescence/SASP memory	DNA damage, oxidative stress, radiotherapy, platinum, gemcitabine, hypoxia	p16, p21, DDR, NF-kappaB, CXCR2 ligands, IL-6, MMPs	Relapse-promoting inflammation, myeloid recruitment, matrix remodeling	Partially reversible with senomorphics; removable with senolytics if safe
Metabolic memory	Nutrient deprivation, hypoxia, oxidative stress, targeted therapy selection	Glycolysis, autophagy, lactate transport, alanine secretion, mitochondrial adaptation	Residual tumor-cell feeding, T-cell dysfunction, redox protection	Potentially reversible but systemic toxicity is a concern
Mechanical ECM memory	Fibrosis, surgery, radiotherapy, cholestasis, tumor contraction	Collagen crosslinking, LOX, integrins, FAK, ROCK, YAP/TAZ	Matrix stiffening, immune exclusion, reduced drug penetration	Potentially reversible through matrix normalization rather than ablation
Vesicle-mediated memory	Therapy-stressed tumor cells, hypoxia, bile-acid stress	Tumor EVs, microRNAs, TGF-beta cargo, metabolic cargo	CAF education, iCAF/vCAF-like induction, cytokine amplification	Potentially targetable through EV-release or uptake pathways
Biliary/cholestatic inflammatory crosstalk	Bile-acid stress, cholestasis, cholangitis, hypoxia, therapy-induced tissue injury	TGF-beta, NF-kappaB, STAT3, oxidative-stress signaling, IL-6/CXCL8, CCL2, YAP/TAZ, ECM-associated feedback	Couples inflammatory CAF activation, SASP signaling, metabolic stress, ECM remodeling and immune suppression in a CCA-specific stromal circuit	Potentially modifiable through bile-acid/inflammation control, stromal normalization or rational stromal-immunotherapy combinations; requires tissue-based validation
Spatial niche memory	Repeated therapy cycles and local tissue injury	CAF-immune-tumor neighborhoods, perivascular organization, collagen tracks	Residual-cell protection and relapse geography	Requires niche-level disruption rather than single-molecule inhibition

This table summarizes candidate memory layers that may contribute to persistent CAF states. These layers are not independent and should not be interpreted as sufficient evidence of therapy-imprinted fibroblast memory in isolation. In CCA, bile-acid stress, cholestasis, hypoxia and chronic biliary inflammation may connect inflammatory, senescent, metabolic and mechanical feedback into a reinforcing stromal circuit, but longitudinal tissue-based validation is required to show exposure history, persistence, measurable signatures, functional consequences and potential reversibility across treatment-naive, post-treatment and recurrent CCA specimens.

CAF, cancer-associated fibroblast; CCA, cholangiocarcinoma; DDR, DNA damage response; ECM, extracellular matrix; EMT, epithelial-mesenchymal transition; FAK, focal adhesion kinase; IL, interleukin; MMP, matrix metalloproteinase; SASP, senescence-associated secretory phenotype; TGF-beta, transforming growth factor-beta.

## 6 Evidence-tiered and testable therapy-imprinted CAF niches in CCA

**Figure 3:**
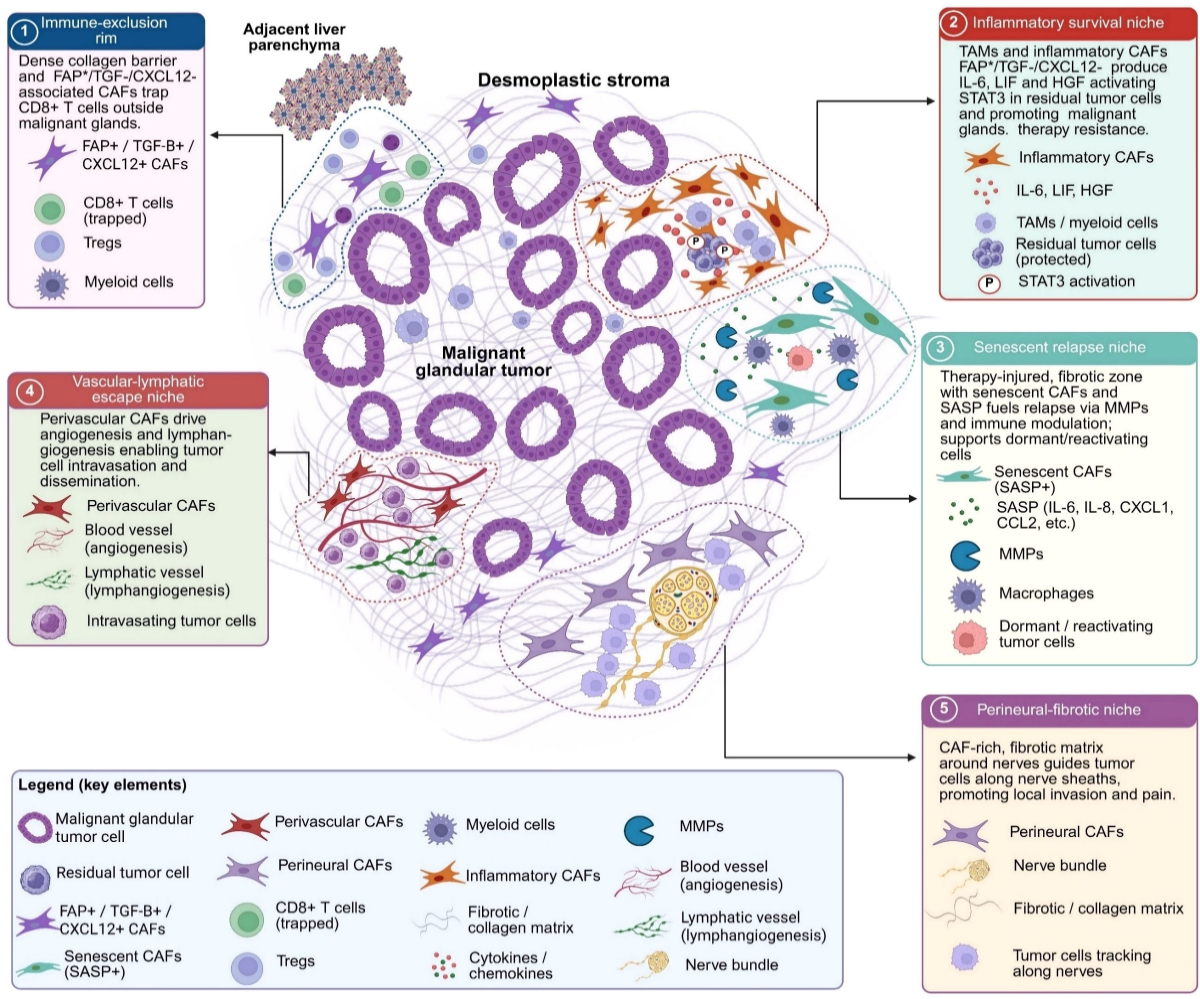
Evidence-tiered and testable therapy-imprinted CAF niches in CCA. This diagram provides a spatial and cellular depiction of the five proposed therapy-imprinted CAF niches. First, the immune-exclusion rim is characterized by dense collagen fibers and FAP/TGF-beta/CXCL12-associated CAF programs at tumor-stroma borders, where CD8+ T cells may be trapped outside malignant glands together with Tregs and myeloid cells. Second, the inflammatory survival niche consists of inflammatory CAFs and tumor-associated macrophages secreting IL-6, LIF, HGF, CXCL12, CCL2 and related cytokines or growth factors, which may activate STAT3 and stress-survival signaling in residual tumor cells. Third, the senescent relapse niche contains therapy-injured fibrotic zones with senescent/SASP-high CAFs releasing IL-6, IL-8, CXCL1, CCL2 and MMPs, thereby promoting myeloid recruitment, matrix remodeling and relapse-permissive inflammation. Fourth, the vascular-lymphatic escape niche shows vCAF-like/perivascular CAF programs near blood vessels and lymphatic structures, supporting angiogenesis, lymphangiogenesis, tumor-cell intravasation and dissemination. Fifth, the perineural-fibrotic niche contains CAF-rich collagen matrix around nerve bundles, where tumor cells may align along nerve-associated stromal tracks and promote local invasion or perineural spread. These niches are not mutually exclusive anatomical compartments but overlapping stromal programs that can be mapped by spatial transcriptomics, multiplex immunofluorescence, imaging mass cytometry, collagen imaging and computational spatial analysis. Each niche is intended as a testable model defined by candidate markers, spatial location, treatment context, CCA-specific evidence and a falsifiable prediction, as summarized in **Table 3**. CCA, cholangiocarcinoma; CAF, cancer-associated fibroblast; FAP, fibroblast activation protein; TGF-beta, transforming growth factor-beta; IL, interleukin; LIF, leukemia inhibitory factor; HGF, hepatocyte growth factor; STAT3, signal transducer and activator of transcription 3; TAM, tumor-associated macrophage; Treg, regulatory T cell; SASP, senescence-associated secretory phenotype; MMP, matrix metalloproteinase; vCAF-like, vascular/perivascular CAF-like program.

Integrating CCA-specific CAF studies with mechanistic evidence from stromal immune exclusion, therapy-induced senescence, angiogenic-lymphangiogenic remodeling and perineural invasion biology, we propose an evidence-tiered five-niche framework for therapy-imprinted CAF memory in CCA. These niches are not presented as equally validated CCA entities. Instead, each niche is defined as a testable spatial model with candidate markers or gene programs, an expected anatomical location, a treatment context, supporting CCA-specific evidence and a falsifiable prediction for validation in paired treatment-naive, post-treatment and recurrent specimens. The first model is the immune-exclusion rim, in which TGF-beta-associated fibroblast programs, FAP/CXCL12 gradients and collagen-rich matrix architecture are predicted to localize at tumor-stroma borders and restrict CD8+ T-cell access to malignant epithelial compartments (78, 96, 125, 133, 134). The second is the inflammatory survival niche, in which IL-6, LIF, HGF, CXCL12, CCL2 and related iCAF-like signals are predicted to protect residual malignant cells through STAT3, growth-factor and stress-survival pathways in post-chemotherapy or post-targeted-therapy tissue (29, 35, 39, 52, 65, 66). The third is the senescent relapse niche, where genotoxic therapy, radiotherapy or chronic biliary injury may induce persistent p16/p21-, CAV1-, SASP- and MMP-associated stromal programs that recruit myeloid cells and remodel matrix around residual tumor foci (34, 42, 46, 79–82). The fourth is the vascular-lymphatic escape niche, in which vCAF-like/perivascular CAF programs may localize around CD31+ blood vessels, LYVE1+/PDPN+ lymphatic structures or invasive perivascular tracks and support angiogenesis, lymphangiogenesis and dissemination through VEGF-, PDGF- and matrix-remodeling pathways (40, 68, 69). The fifth is the perineural-fibrotic niche, in which CAF-rich collagen tracks, Schwann-cell-associated signaling and nerve-adjacent immune remodeling are predicted to preserve invasive residual disease around nerve bundles and anatomically protected tumor borders (77, 78, 130, 135–137). These five niches — immune-exclusion rim, inflammatory survival niche, senescent relapse niche, vascular-lymphatic escape niche, and perineural-fibrotic niche — are proposed as spatially organized stromal programs that can be visualized and tested using multiplex imaging and spatial transcriptomics (**Figure 3**). Recent CCA spatial studies now provide disease-specific support for this framework by mapping leading-edge stromal architecture in iCCA, spatially resolved prognostic microenvironmental programs in iCCA and perineural invasion-associated epithelial-stromal-immune remodeling in dCCA (128–130).

These niches should be interpreted as overlapping stromal programs rather than mutually exclusive anatomical compartments. A post-treatment specimen may contain immune-exclusion and senescent programs simultaneously, or inflammatory and vascular-lymphatic programs within the same invasive region. The practical goal is therefore not simply to name CAF niches, but to determine which spatial program dominates resistance in a given patient, which molecular readout identifies it, and which prediction can be tested experimentally. This framework can be evaluated using paired baseline and progression biopsies, spatial transcriptomics, multiplex immunofluorescence, collagen imaging, computational measurement of T-cell exclusion distance, vessel-CAF proximity analysis, nerve-adjacent CAF mapping and organoid-CAF-immune functional assays, with analyses explicitly separating CCA-validated markers from cross-cancer mechanistic predictions (118, 121–124, 133, 134, 138–143). To make the five-niche framework experimentally testable, **Table 3** summarizes the candidate markers or gene programs, spatial locations, treatment contexts, CCA-specific evidence, testable predictions and suggested validation assays for each proposed niche.

**Table 3:** Evidence-tiered and testable features of the proposed therapy-imprinted CAF niches in CCA.

Niche	Markers, location and treatment context	CCA-specific evidence level	Testable prediction and validation assays
Immune-exclusion rim	Markers/programs: FAP, CXCL12, TGFB1, ACTA2, COL1A1/COL1A2, POSTN, SERPINE1, LOX, FAK/YAP-TAZ activation. Location: tumor-stroma border, invasive margin, collagen-rich rim around malignant ducts, immune-excluded tumor nests. Treatment context: GemCis plus PD-1/PD-L1 blockade, radiotherapy, locoregional therapy, recurrent fibrotic lesions.	Supported by dense CCA desmoplasia, baseline CAF programs and emerging spatial mapping of leading-edge stromal architecture; TGF-beta/CXCL12 immune-exclusion mechanisms are strengthened by cross-cancer evidence.	Prediction: responders and non-responders to chemo-immunotherapy will differ in CD8+ T-cell exclusion distance, FAP/CXCL12/TGF-beta enrichment and collagen barrier organization at tumor borders. Validation: multiplex IF or IMC for CD8/FAP/CXCL12/TGF-beta, collagen SHG, spatial transcriptomics, T-cell exclusion-distance analysis and paired baseline/progression biopsies.
Inflammatory survival niche	Markers/programs: IL6, LIF, HGF, CXCL12, CCL2, CXCL8, CXCL14, STAT3 activation and iCAF-like cytokine program. Location: residual tumor-cell clusters, macrophage-rich stromal regions, post-chemotherapy inflammatory zones and tumor-stroma interface. Treatment context: gemcitabine/cisplatin, FGFR2 inhibition, IDH1 inhibition and chemo-immunotherapy progression.	Supported by CCA CAF-tumor crosstalk, IL-6/HGF-related survival signaling and stromal chemoresistance studies; specific post-treatment spatial persistence requires validation.	Prediction: residual tumor cells adjacent to IL6/LIF/HGF-high CAFs will show increased phospho-STAT3 or stress-survival signaling and relative resistance to chemotherapy or targeted therapy. Validation: ligand-receptor inference, phospho-STAT3 staining, multiplex IF for IL-6/LIF/HGF/CAF markers, organoid-CAF co-culture and perturbation of IL-6/JAK/STAT3 or HGF/MET signaling.
Senescent relapse niche	Markers/programs: p16, p21, CAV1-related patterns, DNA damage-response genes, NF-kappaB, IL6, CXCL8, CCL2, MMPs and SASP score. Location: therapy-injured fibrotic zones, treated margins, hypoxic residual areas and recurrent lesions after genotoxic therapy or radiotherapy. Treatment context: GemCis, radiotherapy, locoregional therapy, surgery-related tissue injury and chronic cholestatic injury.	Supported by CAV1-related CAF senescence in CCA and broader therapy-induced senescence/SASP evidence; longitudinal post-treatment CCA validation remains necessary.	Prediction: post-treatment or recurrent lesions will contain persistent senescent/SASP-high CAF regions associated with myeloid recruitment, matrix remodeling and residual tumor-cell survival. Validation: p16/p21/CAV1 multiplex IF, SASP proteomics, spatial transcriptomics, beta-gal-associated assays where feasible, macrophage proximity analysis and senomorphic or senolytic testing in patient-derived models.
Vascular-lymphatic escape niche	Markers/programs: RGS5, MCAM, PDGFRB, CSPG4, VEGFA, PDGFB/PDGFRB, ANGPT-related programs, MMPs and lymphangiogenic factors. Location: perivascular regions, CD31+ blood vessels, LYVE1+/PDPN+ lymphatic structures and invasive vascular tracks. Treatment context: anti-angiogenic pressure, locoregional therapy, hypoxia after radiotherapy or vascular pruning, and advanced or metastatic disease.	Supported by CCA lymphangiogenesis and stromal dissemination literature; vCAF-like post-treatment evolution needs spatial validation in treated CCA tissues.	Prediction: recurrent or metastatic lesions will show enrichment of vCAF-like/perivascular CAF programs adjacent to blood or lymphatic vessels and increased tumor-cell proximity to vascular escape routes. Validation: spatial mapping of PDGFRB/RGS5/MCAM with CD31, LYVE1 and PDPN, vessel-CAF proximity analysis, perfusion imaging, lymphangiogenesis markers and matched primary-metastatic comparison.
Perineural-fibrotic niche	Markers/programs: COL1A1/COL1A2, POSTN, FAP, ACTA2, MMPs, Schwann-cell-associated signals, nerve-associated ECM programs and LAMB3-DAG1 axis if validated in dCCA context. Location: perineural invasion fronts, nerve bundles, fibrotic tracks surrounding nerves and anatomically protected invasive margins. Treatment context: postoperative recurrence, locoregional therapy, residual disease at invasive or periductal margins, and distal CCA with perineural invasion.	Supported by pathological evidence linking perineural invasion with CCA progression and recent subcellular spatial mapping of distal CCA perineural invasion.	Prediction: recurrent or margin-associated CCA will show CAF-rich collagen tracks and epithelial-stromal-immune remodeling around nerve-associated structures, with tumor cells preferentially aligned along nerve-adjacent stromal pathways. Validation: spatial transcriptomics or multiplex IF around nerve bundles, collagen SHG, nerve marker/CAF co-staining, Schwann-cell proximity analysis and comparison of PNI-positive and PNI-negative regions.

These niches are proposed as evidence-tiered working models rather than fixed anatomical compartments. Candidate markers are not individually diagnostic. A niche should be considered therapy-imprinted only when it is linked to prior exposure, persistence beyond acute injury, measurable molecular or spatial signatures, functional consequences and potential reversibility or targetability.

CAF, cancer-associated fibroblast; CCA, cholangiocarcinoma; DDR, DNA damage response; FAK, focal adhesion kinase; FAP, fibroblast activation protein; GemCis, gemcitabine plus cisplatin; HGF, hepatocyte growth factor; IF, immunofluorescence; IMC, imaging mass cytometry; MMP, matrix metalloproteinase; PD-1, programmed cell death protein 1; PD-L1, programmed death-ligand 1; PNI, perineural invasion; SASP, senescence-associated secretory phenotype; SHG, second harmonic generation; STAT3, signal transducer and activator of transcription 3; TGF-beta, transforming growth factor-beta.

Preclinical CCA data suggest that stromal rewiring can influence immunotherapy response. Laser-activated nanoparticles were reported to remodel the CCA tumor microenvironment and enhance PD-1 blockade-associated T-cell responses, indicating that physical and immune features of CCA stroma can be therapeutically modified (84). This type of evidence, together with CXCL12/FAP and TGF-beta immune-exclusion models, supports the broader concept that CAF niches are dynamic and potentially targetable systems rather than static barriers (85, 144). This evidence-tiered table also clarifies which aspects of the model are supported by CCA-specific observations and which remain cross-cancer mechanistic predictions. The framework can therefore be falsified or refined: a proposed niche should be weakened if paired CCA specimens fail to show the expected spatial pattern, persistence after treatment or functional association with residual disease, immune exclusion or relapse.

## 7 Therapy-imprinted CAFs and immune evasion

CAF-derived chemokine gradients can exclude effector T cells from malignant regions. In pancreatic cancer, targeting CXCL12 from FAP-expressing CAFs synergized with anti-PD-L1 therapy, providing a mechanistic model for testing CXCL12/CXCR4 blockade in CCA immune-exclusion niches (85, 124). TGF-beta-associated stromal programs can attenuate response to PD-L1 blockade by contributing to T-cell exclusion, which is directly relevant to dense CCA stroma (144). TGF-beta can also drive immune evasion in metastatic colon cancer models, strengthening its general role as a stromal immune-suppressive axis (125).

FAP-positive stromal cells can suppress antitumor immunity, and their depletion in mouse models produced strong immune activation, although safety and disease-specific effects require caution (145). Targeting tumor-associated fibroblasts can increase intratumoral drug uptake and improve chemotherapy in experimental models, which supports the rationale for combining stromal modulation with cytotoxic therapy (81). However, immune targeting of FAP can also recognize multipotent bone marrow stromal cells and produce toxicity, emphasizing that FAP-directed strategies need careful design and patient selection (82). CAR-T targeting of FAP-positive stroma has shown antitumor activity in experimental systems, but translation requires attention to on-target off-tumor effects (77). FAP imaging with 68Ga-FAPI PET/CT has shown tracer uptake across many tumor types, including stromal-rich cancers, and may become useful for selecting or monitoring CAF-targeted strategies in CCA (135).

## 8 Treatment-specific routes to relapse

Chemotherapy may imprint CAFs through DNA damage, oxidative stress, senescence and tissue-repair signaling in experimental and cross-cancer contexts, but these changes should be classified as therapy-imprinted fibroblast memory only if they persist beyond acute treatment injury and functionally alter residual tumor-cell survival, immune-cell localization, matrix remodeling or subsequent treatment response. In CCA, current evidence supports stromal crosstalk, CAF-mediated survival signaling and the clinical relevance of relapse after GemCis-based therapy, whereas a fully mapped longitudinal CAF-memory trajectory remains to be established (5, 29, 35, 46, 65–67, 81, 82). In CCA, gemcitabine/cisplatin may reduce tumor burden while leaving residual fibroblast states that secrete survival factors, remodel matrix and interact with suppressive immune cells; this possibility is consistent with known CCA CAF-tumor crosstalk and the clinical pattern of relapse after GemCis-based therapy (5, 29, 65–67). A clinically testable question is whether a post-GemCis CAF signature predicts early relapse, poor response to second-line FOLFOX or limited benefit from subsequent immunotherapy (6, 146).

Chemo-immunotherapy creates a different therapeutic pressure. Gemcitabine/cisplatin plus durvalumab or pembrolizumab has improved outcomes in advanced biliary tract cancer, and recent consensus recommendations now support cisplatin-gemcitabine plus immune checkpoint inhibition as a standard first-line approach for advanced CCA (8, 9, 147). However, many patients still progress, implying that immune activation may be constrained by tumor-intrinsic and microenvironmental barriers. CAF programs that preserve TGF-beta, CXCL12 or collagen-rich immune-exclusion barriers provide a plausible explanation for tumors in which lymphocytes accumulate at the margin but fail to contact malignant ducts (85, 125, 134, 144). This hypothesis should be evaluated in paired biopsies from patients receiving GemCis-ICI regimens using spatial immune monitoring rather than bulk immune-cell abundance alone (138, 146).

Targeted therapies raise an additional issue: stromal bypass. FGFR2 inhibitors and IDH1 inhibitors directly target tumor-cell dependencies, but residual cells may survive through CAF-derived HGF, IL-6, EGF-family ligands, ECM-integrin signaling or metabolic support (10–15, 65, 66). Because acquired-resistance studies in CCA often prioritize tumor-cell mutations, post-FGFR or post-IDH progression biopsies should also be examined for CAF niches that provide adaptive survival cues through growth-factor, mechanical or metabolic pathways (104, 112–115).

Radiotherapy, locoregional therapy and surgery may also imprint fibroblasts through wound-healing, hypoxia, vascular remodeling and matrix deposition (46, 76, 87, 90). Recent clinical studies further illustrate the evolving therapeutic landscape in CCA and BTC. SWOG S1815 evaluated gemcitabine, cisplatin and nab-paclitaxel versus gemcitabine and cisplatin in newly diagnosed advanced BTC, underscoring the continued need to understand why intensified cytotoxic backbones do not uniformly translate into durable disease control (148). In advanced or metastatic intrahepatic CCA after chemotherapy, the CASTLE-01 phase 2 trial suggested that cryoablation followed by sintilimab and lenvatinib can reshape antitumor immunity and tumor-stromal interactions, providing a clinically relevant example of how local therapy, immune checkpoint blockade and anti-angiogenic/targeted therapy may remodel the tumor microenvironment (149). These studies support the importance of embedding stromal and spatial immune biomarkers into future CCA therapeutic trials. Postoperative recurrence may occur in a liver or biliary bed where fibroblasts have been primed by surgical repair, cholangitis, obstruction, ischemia-reperfusion injury and prior systemic therapy. These repair-associated fibroblast responses should be interpreted as therapy-imprinted memory only if they remain detectable after acute wound healing, acquire reproducible stromal or spatial signatures and contribute to recurrence-associated immune or matrix remodeling (41, 76–78, 88–90, 135–137). Perineural invasion is frequently reported in CCA and has been associated with recurrence and poor survival, making nerve-associated fibrotic niches an especially relevant but underexplored post-treatment compartment (140–143). This provides a rationale for collecting recurrent tumors and adjacent liver from patients treated with defined perioperative or locoregional regimens. **Table 4** summarizes the predicted CAF responses, immune consequences, relapse phenotypes and research priorities associated with specific treatment pressures in CCA, including gemcitabine/cisplatin, chemo-immunotherapy, FGFR2 inhibition, IDH1 inhibition, radiotherapy/locoregional therapy, and surgery with biliary injury.

**Table 4:** Treatment pressures and predicted CAF imprinting in CCA.

Treatment pressure	Likely CAF response	Immune consequence	Relapse phenotype	Research priority
Gemcitabine/cisplatin	DNA damage, SASP, iCAF enrichment, ECM repair response	Myeloid recruitment and reduced cytotoxic T-cell function	Early systemic progression or local fibrotic residual disease	Paired baseline/progression biopsy after GemCis
GemCis plus PD-1/PD-L1 blockade	Interferon, TGF-beta and CXCL12 pathway selection	T-cell margin trapping or incomplete tumor-nest infiltration	Primary ICI resistance despite inflammatory signals	Spatial T-cell exclusion metrics in responders vs non-responders
FGFR2 inhibition	Stromal bypass through HGF/IL-6/ECM-integrin programs	Secondary immune-cold resistant niche	Acquired progression without only kinase-domain mutation	Post-FGFR biopsy with ligand-receptor analysis
IDH1 inhibition	Metabolic and epigenetic microenvironment adaptation	Unclear; may involve myeloid and fibroblast reprogramming	Slow progression with stromal survival support	Combine tumor epigenetics with CAF spatial profiling
Radiotherapy/locoregional therapy	Fibrotic wound-healing, vascular remodeling, hypoxia	Hypoxic immune suppression and macrophage recruitment	Local recurrence near treated margins	Map collagen and hypoxia in treated lesions
Surgery and biliary injury	Repair fibroblast activation, cholangitis-associated inflammation	Inflammatory wound bed and immune remodeling	Postoperative or anastomotic-region recurrence	Collect recurrent tumors and adjacent liver after defined perioperative regimens

The CAF responses and relapse phenotypes shown here are proposed working hypotheses. They should not be interpreted as therapy-imprinted fibroblast memory unless linked to prior exposure, persistence beyond acute injury, measurable molecular or spatial signatures, functional consequences and potential reversibility or targetability. CCA-specific studies support the baseline stromal programs, tumor-CAF crosstalk and therapeutic context, whereas related desmoplastic tumor, fibrosis and wound-healing models provide mechanistic support for treatment-induced fibroblast reprogramming. Prospective paired CCA specimens are required to validate these treatment-specific CAF trajectories.

CAF, cancer-associated fibroblast; CCA, cholangiocarcinoma; FGFR, fibroblast growth factor receptor; GemCis, gemcitabine plus cisplatin; ICI, immune checkpoint inhibitor; IDH1, isocitrate dehydrogenase 1; IL, interleukin; MHC, major histocompatibility complex; PD-1, programmed cell death protein 1; PD-L1, programmed death-ligand 1; TGF-beta, transforming growth factor-beta.

## 9 Biomarkers and experimental validation

Biomarker development should integrate molecular markers with spatial metrics. Tissue markers include ACTA2, TAGLN, COL1A1, COL3A1, POSTN and FN1 for myofibroblastic matrix programs; IL6, LIF, CXCL12, CCL2 and HGF for inflammatory survival programs; HLA-DRA and CD74 for antigen-presenting CAF-like states; RGS5, MCAM and PDGFRB for perivascular programs; and FAP, PDPN, SERPINE1, TGFB1 and CXCL12 for immunoregulatory stromal niches (29–32, 38, 40, 52). Senescence-associated biomarker panels should include p16, p21, CAV1-related patterns, SASP cytokines and MMPs, but these markers should be interpreted with spatial and treatment-history context (33, 42, 136, 137). A comprehensive list of biomarkers for therapy-imprinted CAF niches — categorized by class (tissue expression, spatial immune metrics, ECM architecture, senescence/SASP, extracellular vesicle cargo, collagen turnover fragments and imaging) with examples, sample types, clinical questions and limitations, including tissue-specificity and liver-disease confounders — is presented in **Table 5**.

**Table 5:** Biomarkers for therapy-imprinted CAF niches.

Biomarker class	Examples	Sample type	Clinical question	Limitations
Tissue expression	FAP, ACTA2, PDPN, PDGFRB, SERPINE1, TGFB1, CXCL12, IL6, LIF	Resection or biopsy	Which CAF program dominates the tumor?	Bulk expression loses spatial context and cell-source specificity
Spatial immune metrics	CD8 exclusion distance, CAF-macrophage proximity, tumor-CAF contact, perivascular CAF enrichment	Multiplex IF, IMC, spatial transcriptomics	Is the tumor immune-excluded or immune-infiltrated?	Requires standardized region-of-interest selection, cell segmentation, batch correction, deconvolution and external validation before clinical use
ECM architecture	Collagen density, fiber alignment, SHG signal, stiffness, hyaluronan content	FFPE or fresh tissue	Does matrix create a physical therapy barrier?	Technique-dependent, regionally heterogeneous and affected by sampling bias, FFPE processing and background fibrosis
Senescence/SASP	p16, p21, CAV1, IL-6, CXCL8, MMPs, SASP score	Tissue, plasma cytokines	Does prior therapy create a senescent relapse niche?	Senescence markers are context-dependent
Extracellular vesicle cargo	miRNAs, TGF-beta-related cargo, CAF-education signatures	Plasma, bile, urine	Can relapse-prone stromal communication be detected non-invasively?	Cell of origin is difficult to prove; hepatic stellate cells, portal fibroblasts, normal fibroblasts, injured biliary epithelium, chronic hepatitis, cirrhosis, cholangitis and biliary obstruction may generate overlapping or false-positive signals
Collagen turnover fragments	Collagen-derived ECM fragments, hyaluronan-related matrix markers, circulating matrix-remodeling products	Plasma or serum	Is treatment associated with fibrotic matrix remodeling or ECM-rich CAF activation?	Not tumor-specific; chronic liver fibrosis, cirrhosis, cholangitis, biliary obstruction and wound-healing responses are major confounders and require paired tissue or imaging confirmation
Imaging	FAPI PET uptake, stromal radiomics, diffusion/perfusion MRI	Clinical imaging	Can high-stroma patients be selected for CAF-targeted therapy?	Need prospective correlation with tissue and outcome; FAPI uptake and stromal radiomics may be confounded by benign fibrosis, inflammation or wound-healing responses

Biomarkers are grouped by practical sample type and readout. Most require analytical standardization, tissue-source validation and prospective clinical validation before use for treatment selection. Liquid and imaging biomarkers should not be considered CAF-specific unless supported by paired tissue, spatial or longitudinal evidence.

CAF, cancer-associated fibroblast; CCA, cholangiocarcinoma; ECM, extracellular matrix; EV, extracellular vesicle; FAP, fibroblast activation protein; IF, immunofluorescence; IMC, imaging mass cytometry; MRI, magnetic resonance imaging; PET, positron emission tomography; SASP, senescence-associated secretory phenotype; spatial ST, spatial transcriptomics.

Spatial biomarkers may be more predictive than bulk expression because immune exclusion and stromal protection depend on cell positioning. Candidate metrics include CD8+ T-cell distance from malignant glands, CAF-tumor contact frequency, collagen fiber density and alignment, FAP-positive CAF neighborhoods, CAF-macrophage proximity, perivascular CAF enrichment and hypoxic-stromal overlap (134, 138, 150, 151). Blood-based markers should remain exploratory and may include collagen turnover fragments, cytokine panels, extracellular vesicle cargo and circulating fibroblast-associated microRNAs. However, their clinical interpretation requires caution because these signals are not intrinsically CAF-specific or CCA-specific. Extracellular vesicle cargos, circulating miRNAs and collagen-derived fragments may originate from tumor-associated CAFs, hepatic stellate cells, portal fibroblasts, normal wound-healing fibroblasts, inflammatory immune cells or injured biliary epithelium (1, 2, 29, 36, 37, 62, 152). Chronic hepatitis, cirrhosis, cholangitis, biliary obstruction and background liver fibrosis may also generate overlapping stromal and inflammatory signals, increasing the risk of false-positive interpretation (62, 71, 72).

Accordingly, circulating CAF-associated biomarkers should be interpreted as longitudinal within-patient signals rather than stand-alone diagnostic or predictive markers. A clinically useful strategy should combine liquid biopsy readouts with tissue-based spatial validation, liver-function context, imaging findings and assessment of active inflammatory or fibrotic liver disease. For example, an increase in collagen turnover fragments or EV-associated CAF signatures should be linked to tissue evidence of CAF-rich ECM remodeling before being interpreted as therapy-imprinted stromal activation. Similarly, FAPI PET uptake should be correlated with histological and spatial markers because benign fibrosis and inflammation can also produce stromal tracer uptake (43, 83, 107, 146, 152).

**Figure 4:**
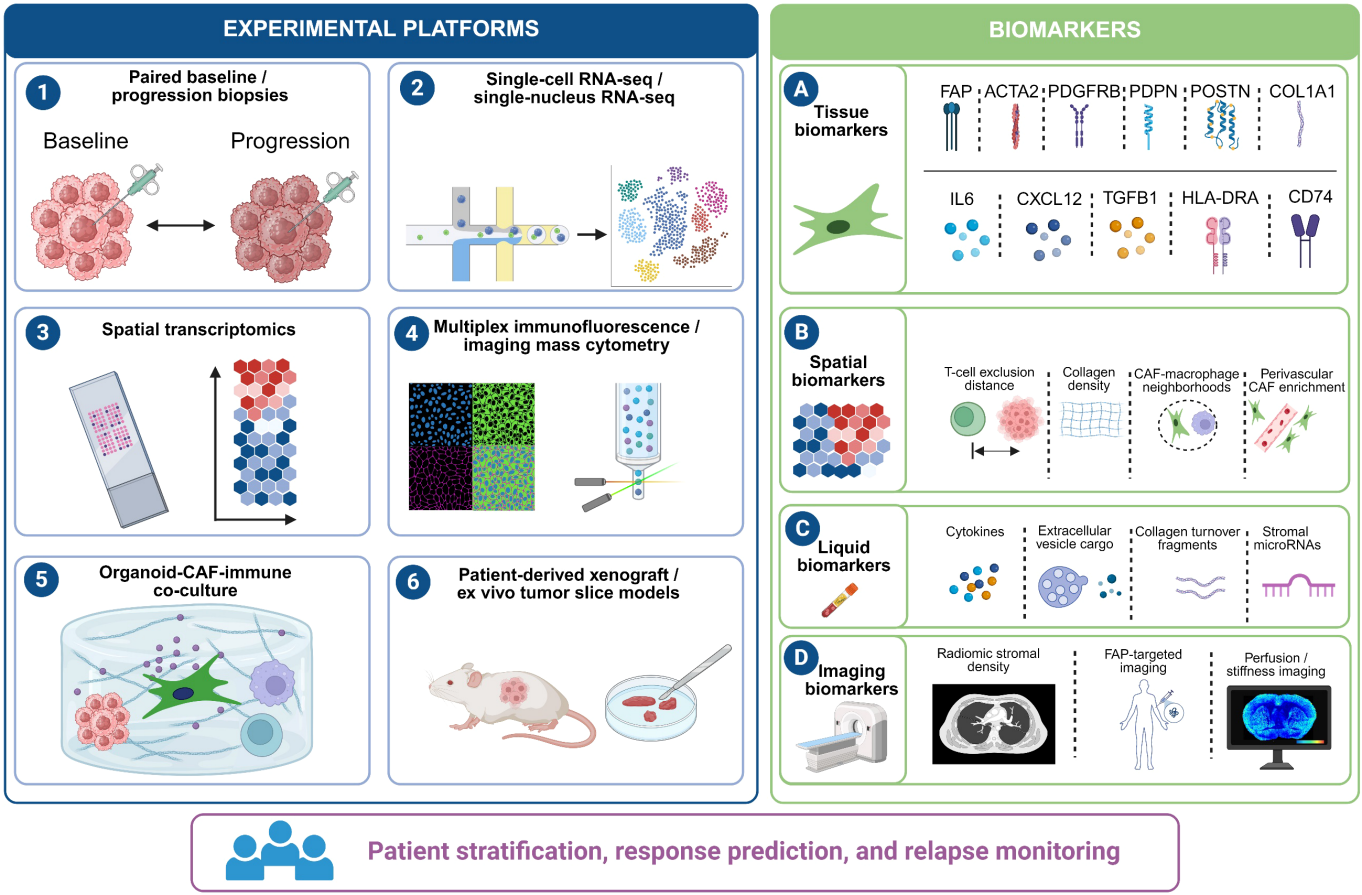
This diagram presents a translational pipeline for validating therapy-imprinted CAF niches in CCA, integrating experimental platforms and biomarker classes. The left side lists key experimental approaches: paired baseline and progression biopsies (pre-treatment, on-treatment, and at relapse) are subjected to single-cell RNA-seq or single-nucleus RNA-seq, spatial transcriptomics, multiplex immunofluorescence or imaging mass cytometry, organoid-CAF-immune co-cultures, patient-derived xenograft (PDX) models, and ex vivo tumor slice models. The right side shows corresponding biomarker categories. Tissue biomarkers include FAP, ACTA2, PDGFRB, PDPN, POSTN, COL1A1, and senescence-associated proteins (p16, p21, CAV1). Spatial biomarkers comprise CD8^+^ T-cell exclusion distance, collagen fiber density and alignment, CAF-macrophage proximity, perivascular CAF enrichment, and hypoxic-stromal overlap. Liquid biomarkers (from plasma, bile or urine) include cytokines, extracellular vesicle cargo (microRNAs, TGF-beta-related proteins), collagen turnover fragments and stromal-associated microRNAs, but these markers require tissue-source validation because hepatic stellate cells, portal fibroblasts, chronic liver fibrosis, cholangitis, biliary obstruction and wound-healing responses can produce overlapping signals. Imaging biomarkers involve radiomic stromal density, FAP-targeted PET (e.g., ^68^Ga-FAPI), and perfusion/stiffness MRI. Integration of these multi-omic, spatial and functional data may support patient stratification, response prediction and relapse monitoring, but clinical translation requires FFPE-compatible validation, cost-aware assay selection, standardized region-of-interest selection, antibody validation, reproducible cell segmentation, locked computational pipelines and prospective outcome correlation. These requirements are essential for converting therapy-imprinted CAF memory from a spatially resolved research framework into a clinically testable biomarker strategy. CCA, cholangiocarcinoma; CAF, cancer-associated fibroblast; FAP, fibroblast activation protein; PET, positron emission tomography; MRI, magnetic resonance imaging; scRNA-seq, single-cell RNA sequencing; PDX, patient-derived xenograft; EV, extracellular vesicle.

Validation should be longitudinal, functional, technically standardized and aligned with the operational definition of therapy-imprinted fibroblast memory. Paired pre-treatment, on-treatment and progression samples should be integrated with scRNA-seq, single-nucleus RNA-seq, spatial transcriptomics, multiplex immunofluorescence and ECM imaging to distinguish baseline CAF heterogeneity from therapy-conditioned CAF evolution (85, 139, 141, 153, 154). Functional relevance should be tested in patient-derived organoids co-cultured with matched CAFs and immune cells, precision-cut tumor slices, PDX models and engineered mouse models (126, 130, 147–149). At the same time, study designs should predefine FFPE quality criteria, minimum tumor area, region-of-interest selection rules, antibody panels, sequencing depth, batch correction, cell segmentation methods, deconvolution algorithms and spatial cutoffs. Without technical standardization, spatial CAF biomarkers will remain difficult to compare across cohorts and platforms. More importantly, without evidence of prior exposure, persistence, measurable signature, functional consequence and potential reversibility or targetability, therapy-imprinted CAF memory will remain a descriptive label rather than a causal or clinically actionable relapse mechanism (121, 123, 124, 140, 143). A systematic experimental pipeline incorporating paired baseline/progression biopsies, single-cell and spatial multiomics, multiplex imaging, organoid-CAF-immune co-cultures, PDX models, multi-class biomarkers and standardized analytical workflows is essential to validate therapy-imprinted CAF niches and guide patient stratification (**Figure 4**).

## 10 Biomarker-guided therapeutic prioritization of therapy-imprinted CAF niches

Therapeutic translation of therapy-imprinted CAF memory should not be framed as indiscriminate CAF depletion or as a broad catalog of stromal targets. A more practical strategy is to match dominant CAF niches with measurable biomarkers, rational combination partners and predefined safety concerns. In this framework, stromal intervention is prioritized only when a CAF program is spatially or functionally linked to immune exclusion, residual tumor-cell survival, impaired drug delivery, relapse-prone ECM remodeling or dissemination. The therapeutic goal is therefore niche rewiring or stromal normalization rather than universal fibroblast ablation (39, 40, 84, 146, 154).

The most actionable first priority is the TGF-beta/ECM-high immune-exclusion niche. This niche should be considered when spatial analysis shows FAP/CXCL12/TGF-beta-enriched CAFs, collagen-rich tumor borders, increased T-cell exclusion distance and low intratumoral CD8+ T-cell penetration. In this setting, TGF-beta-pathway modulation, FAK/YAP-TAZ or mechanotransduction targeting, collagen-remodeling strategies or ECM normalization may be rationally paired with immune checkpoint blockade, but only in biomarker-selected patients and preferably within early-phase trials with mandatory tissue collection (43, 84, 124, 144). Key safety concerns include impaired tissue repair, excessive immune activation, hepatic or biliary inflammation and the risk that broad stromal disruption could remove restraining fibroblast functions.

The second priority is the IL-6/STAT3 inflammatory survival niche. This strategy is most relevant when post-treatment tissue shows IL6/LIF/HGF/CXCL12/CCL2-high iCAF-like programs adjacent to residual tumor-cell clusters, together with phospho-STAT3 activation or stress-survival signaling in malignant cells. In such patients, JAK/STAT3-axis modulation, IL-6-pathway inhibition or HGF/MET-directed strategies may be considered as stromal-sensitizing approaches rather than stand-alone CAF therapies (29, 35, 39, 52, 65, 66, 85). These approaches require caution because inflammatory cytokine pathways also contribute to host defense, liver regeneration and tissue repair; therefore, infection risk, impaired biliary healing, systemic immunosuppression and hepatic toxicity should be prospectively monitored.

The third priority is the senescent/SASP relapse niche. Patients with persistent p16/p21-, CAV1-, SASP- or MMP-high CAF regions after chemotherapy, radiotherapy, locoregional therapy or chronic biliary injury may be considered for senomorphic sequencing, SASP-modulating strategies or carefully designed senolytic/senomorphic combinations before subsequent immunotherapy, but only when tissue biomarkers, spatial analysis and longitudinal blood markers together confirm persistent senescence-associated inflammation rather than nonspecific signals from background liver fibrosis, cholangitis or wound healing (34, 42, 46, 62, 124). Key safety concerns include loss of beneficial wound-healing fibroblasts, exacerbation of liver injury, cytopenia or off-target toxicity depending on the selected senescence-directed agent.

CXCL12/FAP-high immunoregulatory states should be treated as biomarker-defined delivery or imaging opportunities rather than as automatic indications for FAP depletion. CXCR4/CXCL12 blockade, FAP imaging, FAP-targeted delivery or carefully designed FAP-directed immune therapy may be relevant only when FAP expression is spatially linked to immune exclusion, matrix remodeling or stromal relapse niches rather than interpreted as a lineage-defining marker alone (122, 139, 142, 143). Safety concerns are particularly important for FAP-directed approaches because FAP-expressing stromal cells may also participate in tissue repair, vascular support or normal mesenchymal homeostasis.

Other niche-directed approaches should be considered exploratory until stronger CCA-specific evidence is available. Metabolic CAF targeting may be relevant when lactate transport, autophagy, lipid remodeling or redox-adaptation programs are spatially linked to residual tumor-cell survival, but these pathways are shared by malignant cells, hepatocytes and immune cells, creating a narrow therapeutic window (44, 87, 113). Vascular-lymphatic niche targeting may be considered when vCAF-like/perivascular programs are associated with CD31+ vessels, LYVE1+/PDPN+ lymphatic structures or dissemination routes, but anti-angiogenic or lymphangiogenic modulation can worsen hypoxia, tissue ischemia or compensatory invasion (40, 68, 69). Perineural-fibrotic niche targeting remains mainly investigational and should first be validated through nerve-adjacent spatial mapping, collagen imaging and functional models of tumor-nerve-CAF interaction (77, 130, 136). **Table 6** provides a biomarker-guided therapeutic prioritization framework that links dominant CAF niches with required biomarker evidence, prioritized therapeutic logic, rational combination partners, safety concerns and validation requirements.

**Table 6:** Biomarker-guided therapeutic prioritization of therapy-imprinted CAF niches.

Dominant niche or state	Required biomarker evidence and validation	Prioritized therapeutic logic and rational combination	Key safety or feasibility concerns
TGF-beta/ECM-high immune-exclusion niche	FAP/CXCL12/TGF-beta enrichment; collagen-rich tumor border; increased CD8+ T-cell exclusion distance; low intratumoral CD8+ T-cell penetration. Validation requires paired tissue collection, spatial T-cell exclusion analysis, collagen imaging and an FFPE-compatible multiplex panel.	ECM normalization, TGF-beta-pathway modulation, FAK/YAP-TAZ or mechanotransduction targeting to improve immune access. Rationally combined with immune checkpoint blockade, chemotherapy-immunotherapy, radiotherapy or locoregional therapy in biomarker-selected patients.	Impaired tissue repair; excessive immune activation; hepatic or biliary inflammation; potential loss of tumor-restraining stromal functions.
IL-6/STAT3 inflammatory survival niche	IL6/LIF/HGF/CXCL12/CCL2-high iCAF-like programs adjacent to residual tumor cells; phospho-STAT3 or stress-survival signaling in malignant cells. Validation requires multiplex IF for cytokine-high CAFs, phospho-STAT3 staining, ligand-receptor analysis and organoid-CAF perturbation assays.	Cytokine-axis or JAK/STAT3-pathway modulation as stromal sensitization rather than stand-alone CAF therapy. Combined with chemotherapy, targeted therapy or immunotherapy depending on treatment context and dominant resistance pathway.	Infection risk; systemic immunosuppression; impaired liver regeneration; biliary healing complications; hepatic toxicity.
Senescent/SASP relapse niche	Persistent p16/p21-, CAV1-, SASP- or MMP-high CAF regions after therapy; myeloid-cell proximity; exclusion of nonspecific fibrosis or cholangitis. Validation requires p16/p21/CAV1 multiplex IF, SASP proteomics, longitudinal blood-tissue correlation and patient-derived model testing.	Senomorphic sequencing, SASP modulation or carefully selected senolytic/senomorphic combinations. Rationally considered before subsequent immunotherapy, chemotherapy rechallenge or relapse-prevention studies in biomarker-confirmed cases.	Loss of wound-healing fibroblasts; exacerbation of liver injury; cytopenia or off-target toxicity; difficulty distinguishing senescence from background fibrosis.
CXCL12/FAP-high immunoregulatory state	Spatially localized FAP/CXCL12 enrichment linked to immune exclusion, matrix remodeling or stromal relapse niche; FAPI PET or tissue FAP IHC confirmation. Validation requires FAPI PET-tissue correlation, FAP/CXCL12 spatial profiling and co-localization with myCAF/iCAF markers.	FAP imaging, FAP-targeted delivery, CXCR4/CXCL12 blockade or cautious FAP-directed immune therapy. Combined with immune checkpoint blockade, stromal-normalizing therapy or targeted delivery of cytotoxic/immunomodulatory agents.	On-target stromal toxicity; impaired tissue repair; vascular or mesenchymal homeostasis disruption; risk of overinterpreting FAP as a lineage marker.
Metabolic support niche	CAF-associated lactate transport, autophagy, lipid remodeling or redox-adaptation programs spatially linked to residual tumor cells. Validation requires metabolic imaging, metabolomics, spatial transcriptomics and organoid-CAF metabolic perturbation assays.	Metabolic disruption only when CAF-derived metabolic support is functionally demonstrated. Combined with chemotherapy, targeted therapy or immunotherapy depending on the metabolic dependency.	Shared metabolic pathways in tumor cells, hepatocytes and immune cells; narrow therapeutic window; systemic toxicity.
Vascular-lymphatic or perineural-fibrotic escape niche	vCAF-like/perivascular CAF programs near CD31+ vessels or LYVE1+/PDPN+ lymphatics; CAF-rich collagen tracks around nerve bundles. Validation requires vessel-CAF proximity analysis, nerve-adjacent CAF mapping, collagen SHG and comparison of PNI-positive and PNI-negative regions.	Exploratory targeting of vascular, lymphatic, matrix or nerve-associated stromal interactions after spatial validation. Combined with anti-angiogenic or locoregional approaches, matrix normalization or investigational tumor-nerve-CAF targeting.	Hypoxia; ischemia; compensatory invasion; impaired biliary healing; limited CCA-specific therapeutic evidence.

Therapeutic prioritization should be based on biomarker-confirmed dominant niches rather than the presence of single CAF markers alone. The table emphasizes niche rewiring and stromal normalization, not indiscriminate CAF depletion. Several strategies remain hypothesis-generating and require prospective validation in CCA cohorts.

CAF, cancer-associated fibroblast; CCA, cholangiocarcinoma; ECM, extracellular matrix; FAK, focal adhesion kinase; FAP, fibroblast activation protein; FFPE, formalin-fixed paraffin-embedded; HGF, hepatocyte growth factor; IHC, immunohistochemistry; IF, immunofluorescence; IL, interleukin; LIF, leukemia inhibitory factor; MMP, matrix metalloproteinase; PNI, perineural invasion; SASP, senescence-associated secretory phenotype; SHG, second harmonic generation; STAT3, signal transducer and activator of transcription 3; TGF-beta, transforming growth factor-beta.

**Figure 5:**
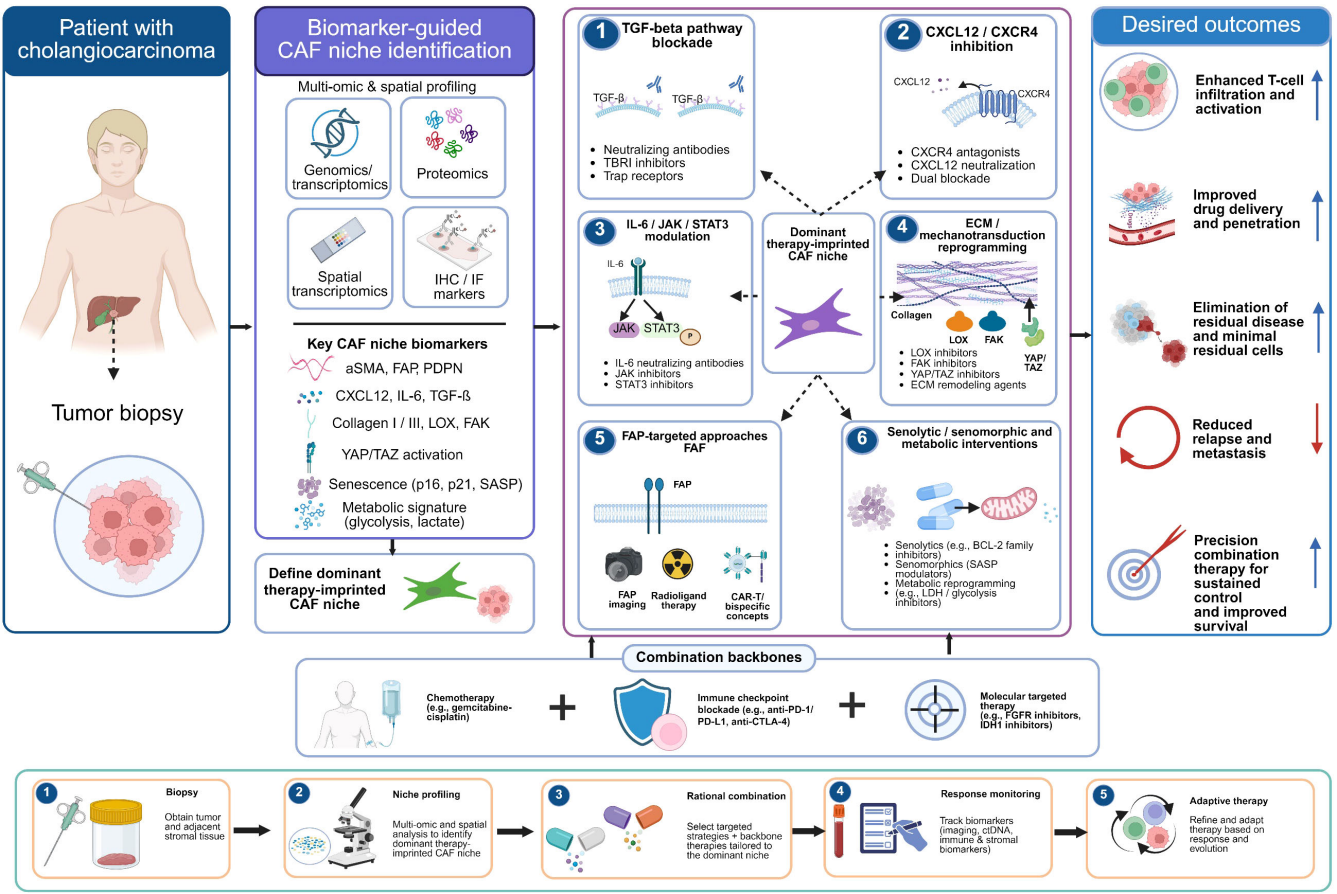
This therapeutic algorithm illustrates a biomarker-driven, niche-specific strategy for rewiring therapy-imprinted CAF niches in CCA. After tumor biopsy, multi-omic and spatial profiling (genomics, transcriptomics, proteomics, spatial transcriptomics, IHC/IF markers) is used to define the dominant therapy-imprinted CAF niche(s) in each patient. Based on the identified niche, rational combination therapies are selected from six intervention categories. For TGF-β/ECM-high immune-exclusion niches, TGF-β pathway blockade (neutralizing antibodies, TβRI inhibitors, trap receptors) or ECM/mechanotransduction reprogramming (LOX inhibitors, FAK inhibitors, YAP/TAZ inhibitors) is combined with immune checkpoint blockade. For CXCL12/FAP-high immunoregulatory niches, CXCR4/CXCL12 inhibition or FAP-targeted approaches (FAP imaging, radioligand therapy, CAR-T or bispecific concepts) are applied. For IL-6/STAT3-high inflammatory survival niches, IL-6/JAK/STAT3 modulation (IL-6 neutralizing antibodies, JAK inhibitors, STAT3 inhibitors) is used together with chemotherapy or targeted therapy. For senescent relapse niches arising after genotoxic therapy, senolytics (e.g., BCL-2 family inhibitors) or senomorphics (SASP modulators) are sequenced after chemotherapy or radiotherapy. For metabolic support niches, interventions targeting lactate transport, autophagy, or amino-acid metabolism are combined with targeted therapy or ICIs. For vascular-lymphatic escape niches, anti-angiogenic or anti-lymphangiogenic strategies are paired with immune or CAF modulation. These niche-specific therapies are combined with backbone treatments (immune checkpoint blockade such as anti-PD-1/PD-L1, or molecular targeted therapy such as FGFR inhibitors and IDH1 inhibitors). Response is monitored using imaging, circulating tumor DNA, and immune/stromal biomarkers, allowing adaptive therapy refinement. The ultimate goals are enhanced T-cell infiltration and activation, improved drug delivery, elimination of residual disease, reduced relapse and metastasis, and durable survival benefit. CCA, cholangiocarcinoma; CAF, cancer-associated fibroblast; ECM, extracellular matrix; FAP, fibroblast activation protein; FAK, focal adhesion kinase; TβRI, transforming growth factor-beta receptor type I; LOX, lysyl oxidase; YAP/TAZ, Yes-associated protein/transcriptional coactivator with PDZ-binding motif; CXCR4, C-X-C chemokine receptor type 4; CAR-T, chimeric antigen receptor T-cell; JAK, Janus kinase; STAT3, signal transducer and activator of transcription 3; SASP, senescence-associated secretory phenotype; ICI, immune checkpoint inhibitor; FGFR, fibroblast growth factor receptor; IDH1, isocitrate dehydrogenase 1.

A practical future trial design would stratify patients by baseline and post-treatment stromal phenotypes and assign stromal interventions only when the dominant niche is biomarker-confirmed. For example, a TGF-beta/ECM-high immune-exclusion profile would require evidence of collagen-rich CAF borders, FAP/CXCL12/TGF-beta enrichment and increased CD8+ T-cell exclusion distance before adding ECM- or TGF-beta-directed modulation to immune checkpoint blockade (8, 9, 43, 84, 144). An IL-6/STAT3 inflammatory survival profile would require IL6/LIF/HGF-high CAF proximity to residual tumor cells and evidence of STAT3 activation before considering cytokine- or JAK/STAT3-axis modulation (29, 35, 39, 66). A senescent/SASP relapse profile would require persistent p16/p21-, CAV1- or SASP-high CAF regions and exclusion of nonspecific fibrosis, cholangitis or wound-healing signals before senomorphic strategies are tested (34, 42, 46, 62, 124). Recent CCA consensus and clinical trial data reinforce the need to embed stromal correlative studies into therapeutic development rather than assuming that improved systemic backbones automatically overcome microenvironmental resistance (147–149). Such approaches require early-phase trials with mandatory tissue collection, predefined spatial biomarkers, FFPE-compatible assay workflows, locked computational pipelines, cost-aware implementation plans and safety stopping rules for stromal-targeted combinations. A biomarker-guided therapeutic algorithm for niche-specific CAF rewiring is summarized in **Figure 5**.

## 11 Conclusions

CCA should be understood as a malignancy in which therapy acts on a multicellular tissue ecosystem, not only on malignant epithelial cells (1, 2, 155). CAFs can acquire post-treatment states through interacting epigenetic, senescent, metabolic, biliary-inflammatory and mechanical memory systems, and these states may persist as spatially organized niches that support immune exclusion, residual disease and treatment-resistant relapse (29, 33, 34, 42, 43, 123, 128, 130). Recent CCA spatial multiomics, consensus and clinical trial studies further support the need to integrate stromal biology into translational trial design (128–130, 147–149). The proposed therapy-imprinted fibroblast memory framework connects desmoplasia, cholestatic injury, wound healing, immune escape and relapse biology into a testable model, but it should be applied only when post-treatment CAF programs show exposure history, persistence, measurable signatures, functional consequences and potential reversibility or targetability. By moving from static CAF taxonomy to longitudinal and spatially resolved CAF evolution, the field may identify stromal biomarkers and biomarker-selected combination therapies capable of converting transient responses into durable disease control, while avoiding indiscriminate CAF depletion and prioritizing niche rewiring, stromal normalization and safety-aware trial design.

## Glossary

ACTA2: actin alpha 2, smooth muscle
AP-1: activator protein-1
apCAF-like: antigen-presentation-like cancer-associated fibroblast program
ARID1A: AT-rich interaction domain 1A
BAP1: BRCA1-associated protein 1
BRAF: B-Raf proto-oncogene, serine/threonine kinase
CAF: cancer-associated fibroblast
CAR-T: chimeric antigen receptor T-cell
CAV1: caveolin-1
CCA: cholangiocarcinoma
CCL2: C-C motif chemokine ligand 2
CD74: cluster of differentiation 74
CD8: cluster of differentiation 8
CODEX: co-detection by indexing
COL1A1: collagen type I alpha 1 chain
COL3A1: collagen type III alpha 1 chain
CTLA-4: cytotoxic T-lymphocyte-associated protein 4
CXCL12: C-X-C motif chemokine ligand 12
CXCR4: C-X-C chemokine receptor type 4
DDR: DNA damage response
DNA: deoxyribonucleic acid
ECM: extracellular matrix
EGFR: epidermal growth factor receptor
EMT: epithelial-mesenchymal transition
ERBB2: erb-b2 receptor tyrosine kinase 2
EV: extracellular vesicle
FAK: focal adhesion kinase
FAP: fibroblast activation protein
FAP-positive CAF state: fibroblast activation protein-positive functional CAF state overlapping with myCAF/iCAF programs
FAPI: fibroblast activation protein inhibitor
FFPE: formalin-fixed, paraffin-embedded
FGFR: fibroblast growth factor receptor
FGFR2: fibroblast growth factor receptor 2
FN1: fibronectin 1
FOLFOX: folinic acid + fluorouracil + oxaliplatin
GemCis: gemcitabine plus cisplatin
HGF: hepatocyte growth factor
HLA-DRA: major histocompatibility complex, class II, DR alpha
iCAF: inflammatory cancer-associated fibroblast
ICI: immune checkpoint inhibitor
IDH1: isocitrate dehydrogenase 1
IF: immunofluorescence
IHC: immunohistochemistry
IL: interleukin
IMC: imaging mass cytometry
JAK: Janus kinase
KRAS: Kirsten rat sarcoma viral oncogene homolog
LDH: lactate dehydrogenase
LIF: leukemia inhibitory factor
LOX: lysyl oxidase
LRRC15: leucine rich repeat containing 15
MCAM: melanoma cell adhesion molecule
MEK: mitogen-activated protein kinase kinase
MHC: major histocompatibility complex
miRNA: microRNA
MMP: matrix metalloproteinase
MRI: magnetic resonance imaging
myCAF: myofibroblastic cancer-associated fibroblast
NF-κB: nuclear factor kappa-B
PAI-1: plasminogen activator inhibitor-1
PD-1: programmed cell death protein 1
PD-L1: programmed death-ligand 1
PDGF: platelet-derived growth factor
PDGFRB: platelet-derived growth factor receptor beta
PDPN: podoplanin
PDX: patient-derived xenograft
PET: positron emission tomography
POSTN: periostin
RNA: ribonucleic acid
ROCK: Rho-associated coiled-coil containing protein kinase
SASP: senescence-associated secretory phenotype
scRNA-seq: single-cell RNA sequencing
SERPINE1: serpin family E member 1
SHG: second harmonic generation
snRNA-seq: single-nucleus RNA sequencing
STAT3: signal transducer and activator of transcription 3
TAM: tumor-associated macrophage
TAZ: transcriptional coactivator with PDZ-binding motif
TβRI: transforming growth factor-beta receptor type I
TGF-β: transforming growth factor-beta
TME: tumor microenvironment
TP53: tumor protein p53
Treg: regulatory T cell
vCAF-like: vascular/perivascular cancer-associated fibroblast-like program
VEGF: vascular endothelial growth factor
YAP: Yes-associated protein
